# AMPK-HIF-1α signaling enhances glucose-derived de novo serine biosynthesis to promote glioblastoma growth

**DOI:** 10.1186/s13046-023-02927-3

**Published:** 2023-12-15

**Authors:** Hye Jin Yun, Min Li, Dong Guo, So Mi Jeon, Su Hwan Park, Je Sun Lim, Su Bin Lee, Rui Liu, Linyong Du, Seok-Ho Kim, Tae Hwan Shin, Seong-il Eyun, Yun-Yong Park, Zhimin Lu, Jong-Ho Lee

**Affiliations:** 1 Department of Health Sciences, The Graduate School of Dong-A University, Busan, 49315 Republic of Korea; 2 Zhejiang Provincial Key Laboratory of Pancreatic Disease, the First Affiliated Hospital, and Institute of Translational Medicine, Zhejiang University School of Medicine, Zhejiang University, Hangzhou, Zhejiang People’s Republic of China; 3 Cancer Center, Zhejiang University, Hangzhou, Zhejiang People’s Republic of China; 4 State Key Laboratory of Oral Diseases, National Clinical Research Center for Oral Diseases, Chinese Academy of Medical Sciences Research Unit of Oral Carcinogenesis and Management, West China Hospital of Stomatology, Sichuan University, Chengdu, Sichuan 610041 People’s Republic of China; 5 Key Laboratory of Laboratory of Medicine, Ministry of Education of China, School of Laboratory Medicine and Life Science, Wenzhou Medical University, Wenzhou, Zhejiang 325000 People’s Republic of China; 6 Department of Biomedical Sciences, Dong-A University, Busan, 49315 Republic of Korea; 7 Department of Life Science, Chung-Ang University, Seoul, 06974 Republic of Korea

**Keywords:** AMPK, HIF-1α, De novo serine synthesis, Serine, Glycine

## Abstract

**Background:**

Cancer cells undergo cellular adaptation through metabolic reprogramming to sustain survival and rapid growth under various stress conditions. However, how brain tumors modulate their metabolic flexibility in the naturally serine/glycine (S/G)-deficient brain microenvironment remain unknown.

**Methods:**

We used a range of primary/stem-like and established glioblastoma (GBM) cell models in vitro and in vivo. To identify the regulatory mechanisms of S/G deprivation-induced metabolic flexibility, we employed high-throughput RNA-sequencing, transcriptomic analysis, metabolic flux analysis, metabolites analysis, chromatin immunoprecipitation (ChIP), luciferase reporter, nuclear fractionation, cycloheximide-chase, and glucose consumption. The clinical significances were analyzed in the genomic database (GSE4290) and in human GBM specimens.

**Results:**

The high-throughput RNA-sequencing and transcriptomic analysis demonstrate that the de novo serine synthesis pathway (SSP) and glycolysis are highly activated in GBM cells under S/G deprivation conditions. Mechanistically, S/G deprivation rapidly induces reactive oxygen species (ROS)-mediated AMP-activated protein kinase (AMPK) activation and AMPK-dependent hypoxia-inducible factor (HIF)-1α stabilization and transactivation. Activated HIF-1α in turn promotes the expression of SSP enzymes phosphoglycerate dehydrogenase (PHGDH), phosphoserine aminotransferase 1 (PSAT1), and phosphoserine phosphatase (PSPH). In addition, the HIF-1α-induced expression of glycolytic genes (*GLUT1*, *GLUT3*, *HK2*, and *PFKFB2*) promotes glucose uptake, glycolysis, and glycolytic flux to fuel SSP, leading to elevated de novo serine and glycine biosynthesis, NADPH/NADP^+^ ratio, and the proliferation and survival of GBM cells. Analyses of human GBM specimens reveal that the levels of overexpressed PHGDH, PSAT1, and PSPH are positively correlated with levels of AMPK T172 phosphorylation and HIF-1α expression and the poor prognosis of GBM patients.

**Conclusion:**

Our findings reveal that metabolic stress-enhanced glucose-derived de novo serine biosynthesis is a critical metabolic feature of GBM cells, and highlight the potential to target SSP for treating human GBM.

**Supplementary Information:**

The online version contains supplementary material available at 10.1186/s13046-023-02927-3.

## Background

Metabolic reprogramming, one of the crucial hallmarks of cancer, provides advantages in cell proliferation and survival for tumor growth [1, 2]. It is well known that the Warburg effect, characterized by enhanced glycolytic flux even in aerobic conditions, is a unique metabolic phenotype in many types of cancer cells [3, 4]. An increased glucose uptake resulting from the Warburg effect supplies cancer cells with energetic and biosynthetic pathways for rapid proliferation or overcoming numerous stresses [2]. Including the Warburg effect, many metabolic pathways are altered or reprogrammed in cancer cells, which are influenced by both genetic alterations (e.g., amplification of metabolic enzymes, activation of putative oncogenes, or loss of tumor suppressor genes) and microenvironmental changes (e.g., altered nutrition, hypoxia, growth factors/cytokines, or infiltrated immune/stromal cells) [5]. In particular, cancer cells adapt their metabolism under the tissue- or organ-specific tumor microenvironment through acquiring metabolic flexibility to support survival and growth, leading to various dependencies and vulnerabilities that could be targeted for therapy [6–8].

The metabolism of serine and glycine is upregulated in many types of human cancers and plays important roles in cancer proliferation and tumor growth [9–16]. As important one-carbon donors to one-carbon metabolism (which comprises two interconnected metabolic cycles, the folate cycle and the methionine cycle), the non-essential amino acids serine and glycine contribute to the one-carbon metabolism-mediated production of cellular building blocks, including nucleic acids, proteins, and lipids, as well as modulation of the NADPH/NADP^+^ ratio and glutathione production to maintain cellular redox balance [17, 18]. Thus, serine and glycine play critical roles in the cellular biosynthesis, metabolic processes, and homeostasis for rapidly proliferating cancer cells. Serine and glycine (which is directly converted from serine by the serine hydroxymethyltransferase (SHMT) 1/2 reaction) can be taken up into cells using a number of different transporters from the extracellular environment or can be synthesized de novo by cells using the serine synthesis pathway (SSP) [17, 18]. The SSP is one of the branches of glycolysis, allowing glucose-derived carbons to be diverted into synthesized serine. The glycolytic intermediate, 3-phosphoglycerate (3-PG) can be converted to serine through three consecutive enzymatic reactions of SSP [17, 18]; Phosphoglycerate dehydrogenase (PHGDH) catalyzes the first step of NAD^+^-dependent oxidation of 3-PG to 3-phosphohydroxypyruvate (3-PHP); subsequently, phosphoserine aminotransferase 1 (PSAT1) converts 3-PHP into 3-phosphoserine (3-PS) in a glutamate-dependent transamination reaction; and finally, serine is then generated from phosphoserine through phosphoserine phosphatase (PSPH)-mediated dephosphorylation. Recent studies have shown that the inhibition of serine biosynthesis and/or exogenous serine supply significantly inhibited the growth of some cancer cells in vitro and in vivo [13, 19], indicating the dependency of rapid proliferating cells on serine. An increased serine biosynthesis is observed in many types of cancer, and has been shown to be required for tumor development. Indeed, PHGDH is frequently overexpressed in estrogen receptor-negative breast cancer, lung adenocarcinoma, and melanoma [9, 10, 20]. Loss of PHGDH or direct blockage of serine biosynthesis via the pharmacological inhibition of PHGDH can inhibit cancer cell proliferation and attenuate tumor growth and metastasis [10, 11, 21, 22]. Hence, serine biosynthesis has emerged as a promising target for therapeutic intervention against cancer.

Differences in amino acid concentrations in the brain microenvironment relative to plasma have been observed. With the exception of glutamine, most amino acid levels are dramatically lower (maximum 100-fold lower) in both the brain interstitial fluid (ISF) and cerebrospinal fluid (CSF), which buffers the brain, than in plasma [21, 23]. In particular, both serine and glycine are among the most depleted amino acids in the brain ISF and the CSF, indicating that the brain is a serine- and glycine-limited microenvironment. Given the brain microenvironment, the growing glioblastoma (GBM) cells, which is a highly malignant primary adult brain tumor with dismal prognosis [24], encounter the limited exogenous serine and glycine, and the cells have to adapt their metabolism to survive and proliferate. However, how GBM cells reprogram their metabolic flexibility to adapt to and overcome these environmental stress conditions for their survival and continuous growth remains unknown.

In this study, we demonstrated that glycolytic metabolism and serine biosynthesis are mainly reprogrammed and activated in GBM cells in response to limited serine and glycine conditions. ROS-AMPK-HIF-1α signaling, activated by serine and glycine deprivation, induces the expression of genes for glucose uptake, glycolysis, and serine synthesis pathway, leading to enforced glucose-derived de novo serine and glycine biosynthesis, which is required for the proliferation and survival of GBM cells and brain tumor growth.

## Materials and methods

### Materials

Rabbit monoclonal antibodies for PHGDH (#66350S, 1:1,000 for immunoblotting, 1:100 for immunohistochemistry), AMPK (#5831S, 1:1,000 for immunoblotting), and p-AMPK (T172, #2535S, 1:1,000 for immunoblotting, 1:100 for immunohistochemistry) were purchased from Cell Signaling Technology (Danvers, MA). Rabbit monoclonal antibodies for PSAT1 (#ab96136, 1:1,000 for immunoblotting, 1:100 for immunohistochemistry) and HIF-1α (#ab51608, 1:1,000 for immunoblotting, 1:100 for immunohistochemistry) were purchased from Abcam (Cambridge, MA). Rabbit monoclonal antibody for PSPH (#13,503-R001, 1:1,000 for immunoblotting, 1:100 for immunohistochemistry) was purchased from Sino Biological (China). Mouse monoclonal antibody for tubulin (#T4026-100UL, 1:1,000 for immunoblotting) was purchased from Sigma-Aldrich (St. Louis, MO). Mouse monoclonal antibodies for lamin B1 (#sc-374015, 1:1,000 for immunoblotting) was purchased from Santa Cruz Biotechnology (Santa Cruz, CA). PX-478 (#S7612) was purchased from Selleck Chemicals (Houston, TX). Cycloheximide (CHX; S1988), Compound C (#171,260), 2-Deoxy-D-glucose (2-DG) (#D6134), Cobalt (II) chloride (CoCl_2_) (#60,818), L-Serine (#S4311), Glycine (#G8790), and Sarcosine (glycine transporter I inhibitor, #131,776) were purchased from Sigma Aldrich (St. Louis, MO). 4-Fluoro-L-2-phenylglycine (D-Serine transport inhibitor, #F0862) was purchased from Tokyo Chemical Industry (Japan). [U-^13^C_6_] glucose (#110,187–42-3) was purchased from Cambridge Isotope Laboratory (Tewksbury, MA).

### Cell culture 

The U87MG and U373MG GBM cells were purchased from the Korean Cell Line Bank (KCLB; Seoul, Republic of Korea). Normal human astrocytes (NHA) and GBM cells, including LN18, T98G, A172, and LN229, were kindly provided by Dr. Hyunggee Kim (Korea University, Seoul, Republic of Korea). The U251 GBM cells were kindly provided by Dr. In Ah Kim (Seoul National University, Seoul, Republic of Korea). All cells were authenticated and routinely tested for mycoplasma. NHA were maintained in Astrocyte medium (#1801, Scien-Cell, Carlsbad, CA). GBM cells were maintained in Dulbecco’s modified Eagle’s medium (DMEM) (#LM001-05, Welgene, Republic of Korea) supplemented with 10% fetal bovine serum (#S001-04, Welgene, Republic of Korea) and 1% Penicillin/Streptomycin (#PS-B, Capricorn Scientific, Germany). For all serine and glycine-deprivation experiments, cells were cultured in customized serine/glycine-deprived medium (#LM001-229, Welgene, Republic of Korea) supplemented with or without dialyzed fetal bovine serum (#26,400,044, Gibco, Gaithersburg, MD). Glioma stem cells (GSCs) originally isolated from human GBM specimens of patients undergoing surgery [25, 26] have been studied by several other groups [27, 28]. They were kindly provided by Dr. Yong Tae Kwon’s group (Seoul National University, Seoul, Republic of Korea). GSCs were maintained in Neurobasal Plus Medium (#A3582901, ThermoFisher; Pittsburgh, PA) supplemented with 2% B-27 (minus vitamin A; #12,857,010, ThermoFisher, Pittsburgh, PA), EGF (20 ng/mL) (#AF100-15, PeproTech, Seoul, Republic of Korea), and FGF (20 ng/mL) (#100-18B, PeproTech, Seoul, Republic of Korea).

### RNA sequencing

The 5 × 10^6^ U87MG cells were seeded in 10 cm dish. After culturing serine/glycine-containing complete media or equivalent media lacking serine/glycine for 24 h, total RNA was extracted. RNA sequencing and library construction were performed according to the QuantSeq 3’ mRNA-Seq Library Prep Kit (#015.2 × 96, Lexogen, Inc., Austria).

### Gene Ontology (GO) and pathway analyses

Enrichr was utilized for functional enrichment analysis of GO annotations database [29], while PANTHER Pathway was used for ontology-based pathway analysis to identify significant biological pathways [30]. Ingenuity Pathway Analysis (IPA) bioinformatics software (Qiagen, Valencia, CA, USA) was employed for network analysis to investigate the relationship between the differentially expressed genes (DEGs) and biological functions [31]. A 1.3-fold change with *P*-value less than 0.05 for the differences in gene expression was used as the cut-off value for determining significant changes in gene expression levels.

### Quantitative real-time PCR analysis

Total RNA was isolated with TRIsure reagent (#BIO-38033, Bioline, UK), according to the manufacturer’s instructions. Equal amount of total RNA was then used for cDNA synthesis using PrimeScript™ RT Master Mix (Perfect Real Time) (#RR036A, Takara, Japan). Real-time PCR was performed on an ABI Prism 7500 sequence detection system using a SYBR® Green PCR Master Mix (#43–091-55, Applied Biosystems; Foster City, CA), following the manufacturer’s protocols. The ABI 7500 sequence detector was programmed with the following PCR conditions: 40 cycles of 15 s denaturation at 95°C, and 1 min amplification at 60°C. The relative differences of PCR results was evaluated using the comparative cycle threshold (CT) method [32]. All reactions were run in triplicate, and normalized to the housekeeping gene *GAPDH*. The following primer pairs were used for quantitative real time-PCR: *PHGDH*, 5’- GCCCTTACCAGTGCCTTCTC -3’ (forward) and 5’-GACAATGACTGCGGGGCTTA -3’ (reverse); *PSAT1*, 5’-GGCCAGTTCAGTGCTGTCC -3’ (forward) and 5’- GCTCCTGTCACCACATAGTCA -3’ (reverse); *PSPH*, 5’-AAATCTGTGGCGTTGAGGAC-3’ (forward) and 5’-ACCTGAACATTTCGCTCCTG-3’ (reverse); *GLUT1*, 5’-TCAACACGGCCTTCACTG-3’ (forward) and 5’-CACGATGCTCAGATAGGACATC-3’ (reverse); *GLUT3*, 5’- TCCCCTCCGCTGCTCACTATTT-3’ (forward) and 5’-ATCTCCATGACGCCGTCCTTTC-3’ (reverse); *HK2*, 5’-AACAGCCTGGACGAGAGCATC-3’ (forward) and 5’-AGGTCAAACTCCTCTCGCCG-3’ (reverse); *PFKFB2*, 5’-AGTCCTACGACTTCTTTCGGC-3’ (forward) and 5’-TCTCCTCAGTGAGATACGCCT-3’ (reverse); *HIF-1α*, 5’-CATAAAGTCTGCAACATGGAAGGT-3’ (forward) and 5’-ATTTGATGGGTGAGGAATGGGTT-3’ (reverse); *GAPDH*, 5’-GCATCTTCTTTTGCGTCG-3’ (forward) and 5’-TGTAAACCATTGTAGTTGAGGT-3’ (reverse).

### Immunoblot analysis

Extraction of proteins from cultured cells was performed using a lysis buffer (50 mM Tris–HCl [pH 7.5], 0.1% sodium dodecyl sulfate (SDS), 1% Triton X-100, 150 mM NaCl, 1 mM DTT, 0.5 mM EDTA, 100 µM sodium orthovanadate, 100 µM sodium pyrophosphate, 1 mM sodium fluoride, and proteinase inhibitor cocktail). The cell extracts were centrifuged at 15,000 rpm (at 4℃ for 15 min), and protein concentrations of cell lysates were determined using the DC protein assay Kit (#5,000,112, Bio-Rad, Hercules, CA). Equal amounts of lysates were resolved by SDS- polyacrylamide gel electrophoresis (PAGE), and were then transferred to a nitrocellulose membrane. The membrane was blocked with 5% skim milk in TBST at room temperature (RT) for 30 min, and next incubated with the indicated antibodies at 4℃ overnight. The blots were then incubated with horseradish peroxidase-conjugated secondary antibodies (anti-rabbit (#NA934V, Sigma Aldrich, St. Louis, MO) or anti-mouse (#NA931V, Sigma Aldrich, MO) at RT for 2 h. Band intensity was quantified using ImageJ 1.53e software (National Institutes of Health). Each experiment was repeated at least three times. Figure S7 of the SI displays full scans of the original immunoblots.

### Nuclear fractionation

Lysis of membrane from cultured cells was performed using a 0.1% NP-40. The cell suspension was centrifuged at 1,000 × *g* (at 4℃ for 5 min). Extraction of nuclear contents from pellet was performed using a radioimmunoprecipitation assay (RIPA) buffer (25 mM Tris–HCl [pH 7.4], 150 mM NaCl, 0.1% SDS, 0.5% sodium deoxycholate, 1% NP-40, proteinase inhibitor cocktail). Nuclear concentrations of cell lysates were determined using the DC protein assay Kit (#5,000,112, Bio-Rad, Hercules, CA). Nuclear and cytosolic proteins were used in immunoblot analyses. Each experiment was repeated at least three times.

### Measurements of glucose consumption, and ATP and AMP levels

The culture medium was collected for measurement of glucose concentrations [33], and the remained cells were measured for adenosine triphosphate (ATP) or adenosine monophosphate (AMP) levels. Glucose levels were determined using a Glucose (GO) Assay Kit (#GAGO20, Sigma, St. Louis, MO). Glucose consumption was the difference in glucose concentration from the collected culture medium between experimental conditions. Absorbance was recorded at 540 nm at RT in a 96-well plate. All results were normalized to the final cell number. The ATP or AMP levels were assessed using an ATP Colorimetric/Fluorometric Assay Kit (#354, BioVision, Milpitas, CA) or an AMP Colorimetric Assay Kit, respectively (#229, BioVision, Milpitas, CA). The reaction was performed using a cell lysate in 100 µL of reaction buffer, which was prepared according to the corresponding assay kit instructions. Absorbance was recorded at 570 nm at RT in a 96-well plate.

### Measurements of intracellular serine and glycine levels

Serine or glycine levels were assessed using a DL-Serine Assay Kit or a Glycine Assay Kit (BioVision, Milpitas, CA), respectively. The reaction was performed using a cell lysate in 100 µL of reaction buffer, which was prepared according to the corresponding assay kit instructions, and the fluorescence was measured at Ex/Em = 535/587 nm in endpoint mode.

### Measurement of the NADPH/NADP^+^ ratio and ROS levels

The NADPH/NADP^+^ ratio was assessed using a NADP/NADPH Quantification Colorimetric Kit (#K347, BioVision, Milpitas, CA). The reaction was performed using a cell lysate in 800 μL of reaction buffer, which was prepared according to the NADP/NADPH Quantification Colorimetric Kit instructions. Absorbance was recorded at 450 nm at RT in a 96-well plate. ROS levels were assessed using a Reactive Oxygen Species (ROS) Detection Assay Kit (#K936, BioVision, Milpitas, CA). The reaction was performed according to the assay kit instructions.

### siRNA and shRNA transfection

AMPKα1/2 siRNA (#sc-45312) was purchased from Santa Cruz (Dallas, TX). HIF-1α siRNA (#104,730,345) was purchased from Integrated DNA Technologies (Coralville, IA). The pLKO.1-puro lentiviral vectors encoding shRNA targeting GFP, PHGDH (#TRCN0000028532), PSAT1 (#TRCN0000035268), PSPH (#TRCN000002795), or HIF-1α were purchased from Sigma Aldrich (St. Louis, MO). Cells were plated at a density of 4 × 10^5^ per 60 mm dish 18 h before transfection. Transfection of siRNAs was performed using Lipofectamine™ RNAiMAX transfection reagent (#13,778,150, ThermoFisher, Pittsburgh, PA), according to the manufacturer’s instructions. Transfection of shRNAs was performed using Lipofectamine^2000^ transfection reagent (#11,668,027, ThermoFisher, Pittsburgh, PA), according to the manufacturer’s instructions.

### Luciferase reporter assay

The tumor cells were co-transfected with a pGL3 empty vector or a pGL3-HRE-luciferase plasmid containing five copies of HREs (as an inner control that contained Renilla luciferase sequences (#E2231, Promega, Madison, WI)) using Lipofectamine^2000^ transfection reagent according to the manufacturer’s instructions, and then grown under different experimental conditions. After incubation, firefly and Renilla luciferase activities were measured using a Dual-Luciferase® Reporter Assay System (#E1910, Promega; Madison, WI), and the ratio of firefly/Renilla luciferase was determined [34, 35].

### Chromatin immunoprecipitation (ChIP) assay

A ChIP assay was performed using a SimpleChIP Enzymatic Chromatin IP kit (#9003S, Cell Signaling Technology, Danvers, MS) [36]. Chromatin prepared from 2 × 10^6^ cells (in a 10 cm dish) was used to determine the total DNA input, and then incubated overnight with PHGDH, PSAT1, or PSPH antibodies, or with normal mouse IgG, at 4℃ overnight. Immunoprecipitated chromatin was detected using real-time PCR. The PCR primer sequences for the *HIF-1α* promoter were *PHGDH*, 5’-GCTTCTGATTCTAGGTGACTT-3’ (forward) and 5’-ACGGGATGTCAGTGTGGTTTA-3’ (reverse); *PSAT1*, 5’-GGAGAATCAGCGACTTTAAAGG-3’ (forward) and 5’-TTGGAAGCGCAGGATGAAGAA-3’ (reverse), *PSPH*, 5’-GCACTCAGCATCGTTTCCTTT-3’ (forward) and 5’-TACATCTTCATGGTGCCCTTG-3’ (reverse).

### Metabolic flux analysis using [U-^13^C_6_]-glucose by LC–MS

Two million U87MG cells were seeded in 10 cm plates in triplicate and cultured in experimental medium with [U-^13^C_6_]-glucose for 12 h. Samples were thawed in an ice bath to reduce sample degradation before processing, and then 400 μL of 80% methanol solution was added to each tube of cell samples, and the cells sonicated. The samples were centrifuged at 18,000 × *g* for 15 min at 4°C, and the supernatant was then collected. Samples were reconstituted in 80% methanol solution, awaiting LC–MS analysis. The metabolites were analyzed by ultrahigh-pressure liquid chromatography–triple quadrupole mass spectrometry (ACQUITY UPLC-Xevo TQ-S, Waters Corp., Milford, MA, USA). For data processing, the raw data files generated by UPLC-MS/MS were processed using MassLynx software (v 4.1, Waters Corp., Milford, MA, USA) for the peak extraction, integration, identification, and quantification of each metabolite. R language (v4.1.1) was used for subsequent statistical analysis.

### Cell proliferation assay

A total of 4 × 10^4^ cells were plated and counted at day (d) (2, 4, and 6) after seeding in DMEM supplemented with 10% fetal bovine serum, or customized serine/glycine-deprived DMEM supplemented with 10% dialyzed fetal bovine serum. The cells were trypsinized and counted using a hemocytometer.

### Annexin V − FITC staining

Apoptosis was determined using an Annexin V staining. Cells were seeded in 12-well plate, stained with Annexin V − FITC (#K101, Biovision, Milpitas, CA) to the manufacturer’s instruction, and then analyzed under a fluorescence microscopy (Nikon, Japan).

### Intracranial implantation of GSCs in mice and histologic evaluation

We injected GSCs (XO6) with or without modulation of PHGDH, PSAT1, or PSPH expression, intracranially into 4-week-old male athymic nude mice (five mice/group), as described previously [37]. The mice were euthanized 21 d after the GSCs were injected. The brain of each mouse was harvested, fixed in 4% formaldehyde in PBS, and embedded in paraffin. After that, histological Sects. (5 μm) were prepared. The sections were stained with Mayer’s hematoxylin (#HK100, Biogenex Laboratories, San Ramon, CA), and subsequently with eosin (H&E) (Biogenex Laboratories, San Ramon, CA). Afterward, the slides were mounted with Universal Mount (Research Genetics Huntsville, AL). Tumor formation and phenotype were determined by histological analysis of H&E-stained sections. Tumor volume was calculated by the formula 0.5 × L × W^2^ (L, length; W, width). All the mice were housed in the Dong-A University animal facility, and all experiments were performed in accordance with relevant institutional and national guidelines. All animal procedures and maintenance conditions were approved by the Dong-A University Institutional Animal Care and Use Committee.

### Terminal deoxynucleotidyl transferase dUTP nick end labeling (TUNEL) assay of intracranial tumors

Apoptotic cells were determined by the DeadEnd™ Colorimetric TUNEL System (#G7360, Promega, Madison, WI), according to the manufacturer’s instruction. Briefly, the tumor sections described above were treated with the equilibration buffer, and incubated for 10 min, followed by 10 min incubation in 20 µg/mL proteinase K solution. The sections were washed in PBS, and incubated with TdT enzyme at 37°C for 1 h in a humidified chamber to incorporate biotinylated nucleotides at the 3′-OH ends of DNA. The slides were incubated in horseradish peroxidase-labeled streptavidin to bind the biotinylated nucleotides, followed by detection with a chromogen 3,3′-diaminobenzidine (DAB) (DAB Substrate Kit, #SK-4100; Vector Laboratories).

### Immunohistochemical (IHC) analysis and scoring

IHC analyses were conducted using paraffin-embedded tissue sections. The expression of PHGDH, PSAT1, PSPH, and Ki-67 was detected with a VECTASTAIN Elite ABC kit (#PK-6200, Vector Laboratories, Burlingame, CA); tissue sections were then incubated with 3,3′-diaminobenzidine (#SK-4100, Vector Laboratories, Burlingame, CA), and the nuclei were stained with hematoxylin. Six randomly chosen fields per slide were analyzed and averaged.

The human GBM samples and clinical information were from the First Affiliated Hospital, Zhejiang University School of Medicine, China. This study was approved by the Ethics Committee of Zhejiang University School of Medicine (China), and written informed consents were obtained from all patients. The tissue sections from 50 paraffin-embedded human GBM specimens were stained with antibodies against p-AMPK (T172), HIF-1α, PHGDH, PSAT1, PSPH or non-specific immunoglobulin as a negative control. We quantitatively scored the tissue sections according to the percentage of positive cells and staining intensity, as previously defined [37]. We assigned the following proportion scores: (0, 1, 2, 3, 4, or 5) if (0, (0.1 to 1), (1.1 to 10), (11 to 30), (31 to 70), or (71 to 100)) %, respectively, of the tumor cells showing positive staining. We rated the intensity of staining on a scale of (0 to 3): 0, negative; 1, weak; 2, moderate; and 3, strong. We then combined the proportion and intensity scores to obtain a total score (range, (0 − 8)), as previously described [37].

### Genomic data analysis

Genomic datasets were downloaded from The Gene Expression Omnibus (GEO) database (https://www.ncbi.nlm.nih.gov/geo) and processed using R-package. Correlation analysis between two genes was performed with *Pearson*’s correlation analysis. *P*-value indicates the significance of correlation.

### Tissue microarray analysis

A paraffin-embedded GBM tissue microarray was obtained from US Biomax (#GL722a, Rockville, MD), and all tissues are collected under the highest ethical standards with the donor being completely informed, and with their written consent. IHC analyses of PHGDH, PSAT1, and PSPH expression were performed according to the protocol described above.

### Statistical analysis

All quantitative data are presented as the mean ± SD of at least three independent experiments. A 2-group comparison was conducted using the 2-sided, 2-sample Student’s t-test. A simultaneous comparison of > 2 groups was conducted using one-way ANOVA, followed by Tukey’s post hoc tests. The SPSS statistical package (version 12; SPSS Inc., Chicago, IL) was used for the analyses. Values of *P* < 0.05 were considered to indicate statistically significant differences.

## Results

### Genes in glycolysis and de novo serine biosynthesis are highly expressed in GBM cells upon serine/glycine deprivation

**Fig. 1:**
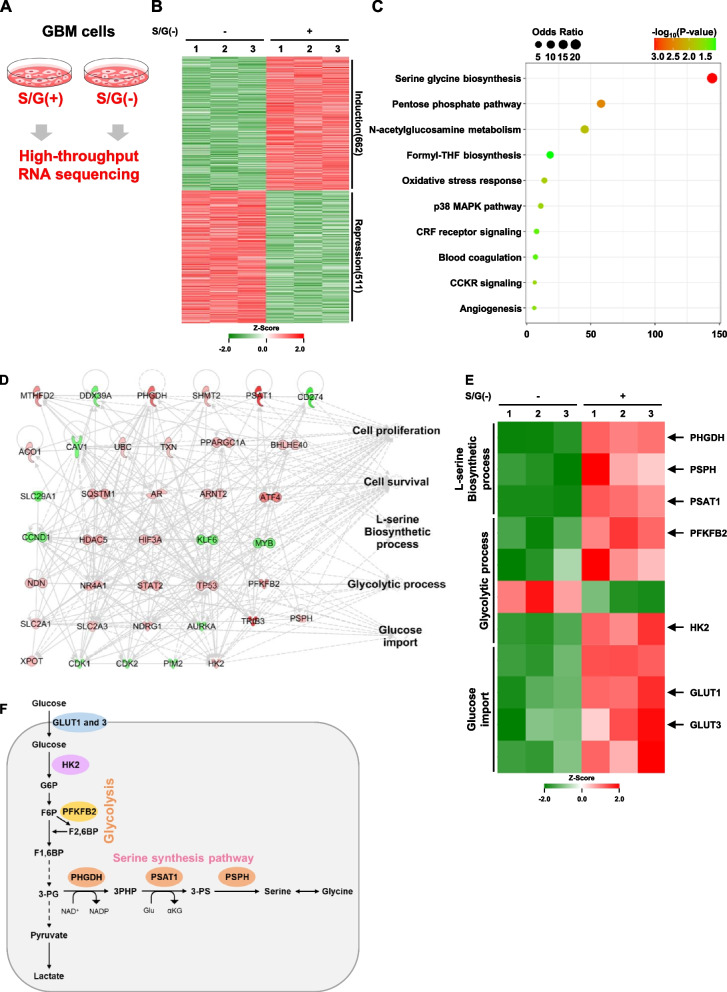
Genes in glycolysis and de novo serine biosynthesis are highly expressed in GBM cells upon serine/glycine deprivation. **A** U87MG cells were cultured with serine/glycine-containing complete media (S/G( +)) or equivalent media lacking serine/glycine (S/G(-)) for 24 h. **B** Heatmap presentation of 1,173 genes (fold change ≥ 1.3 and ≤  − 1.3; *P* < 0.05) in U87MG cells cultured as in (A). **C** Bubble plot for ontology-based pathway enrichment analysis for DEGs of U87MG cells in S/G-deprived U87MG cells, compared to control. Enrichment score is calculated with odds ratio and *P*-value and used to rank the pathways. Bubble size indicates odds ratio and is used to assess the strength of association between DEGs and the pathway. Color indicates − log10 (*P*-value) for determination of the representation significance. THF, tetrahydrofolate; CRF, Corticotropin releasing factor; CCKR, cholecystokinin receptor. **D** Analysis of trimmed DEGs network with biological functions, including cell proliferation, cell survival, L-serine biosynthetic process, glycolytic process, and glucose import, using IPA in S/G-deprived U87MG cells, compared to control. The analysis involved a fold change cut-off value of ± 1.3. Red and green colors indicate genes that were upregulated and downregulated, respectively, compared to the control. Figure S1A of the SI provides details of shape, which originate from Ingenuity Systems (http://www.ingenuity.com). **E** Heatmap presentation for gene expression profiles in the L-serine biosynthetic process, glycolytic process, and glucose import. **F** Schematic of the glucose-derived serine synthesis pathway

To determine whether GBM cells have altered gene expression profiles under the limited serine and glycine conditions of the extracellular environment, we subjected control- or serine/glycine (S/G)-deprived U87MG GBM cells to high-throughput RNA sequencing (Fig. 1A). A total of 25,737 genes were detected in these analyses. Among them, 1,173 genes were differentially expressed (fold change ≥ 1.3 and ≤  − 1.3 with *P*-value less than 0.05 for the differences); expression of 662 genes were upregulated, while expression of 511 genes were downregulated in response to S/G deprivation in U87MG cells (Fig. 1B). Functional enrichment analysis with gene ontology (GO) annotations for biological processes showed that several cellular biological processes, including starvation, apoptosis, molecular transports, and serine family amino acid biosynthesis process, were highly ranked (Table S1of the SI). Moreover, in the analyses with GO annotations for molecular function, the differentially expressed genes (DEGs) were highly related to transporter activities as 5 of the top 10 enriched molecular function categories (Table S2 of the SI). In the analyses with GO annotations for cellular component, DEGs were highly related to lysosome and autophagosome as 8 of the top 10 enriched cellular component categories (Table S3 of the SI). In addition, ontology-based pathway enrichment analysis revealed that the DEGs were highly associated with serine/glycine biosynthesis, which was the top-ranked enriched pathway (Fig. 1C). We next analyzed the DEGs network of S/G-deprived U87MG cells by Ingenuity Pathway Analysis (IPA) [31], and found the major cluster of the genes network (Fig. S1A of the SI). The network was further trimmed by providing higher relevance among the genes. We observed that the gene network was highly associated with representative biological functions, such as cell proliferation, cell survival, and glucose metabolism disorder (Fig. S1B and Table S4 of the SI), in which the glucose metabolism disorder was subdivided into glycolytic process and glucose import (Fig. 1D). Intriguingly, we also found that L-serine biosynthetic process-related genes (i.e., *PHGDH, PSAT1, PSPH,* and *SHMT2*), which were top-ranked in the ontology-based pathway enrichment analysis, were included and associated in the network (Fig. 1D and Fig. S1C of the SI). As the glucose-utilized glycolytic pathway is directly linked to the de novo serine synthesis pathway (SSP), we focused on the gene sets associated with glycolytic metabolism and SSP. Of these genes, multiple genes associated with glucose transporters (i.e., *Glucose transporter 1 and 3 (GLUT1* and *GLUT 3)*), glycolytic pathway (i.e., *hexokinase 2 (HK2)* and *6-phosphofructo-2-kinase/fructose-2,6-biphosphatase 2 (PFKFB2*)), and SSP (i.e., *PHGDH*, *PSAT1*, and *PSPH*) were specifically upregulated in response to S/G deprivation (Fig. 1E and F). These results demonstrate that the pathways in glucose uptake, glycolytic process, and de novo serine biosynthesis are highly upregulated in GBM cells in response to serine/glycine deprivation.

### Serine/glycine deprivation induces glucose uptake, glycolytic flux, and de novo serine biosynthesis

**Fig. 2:**
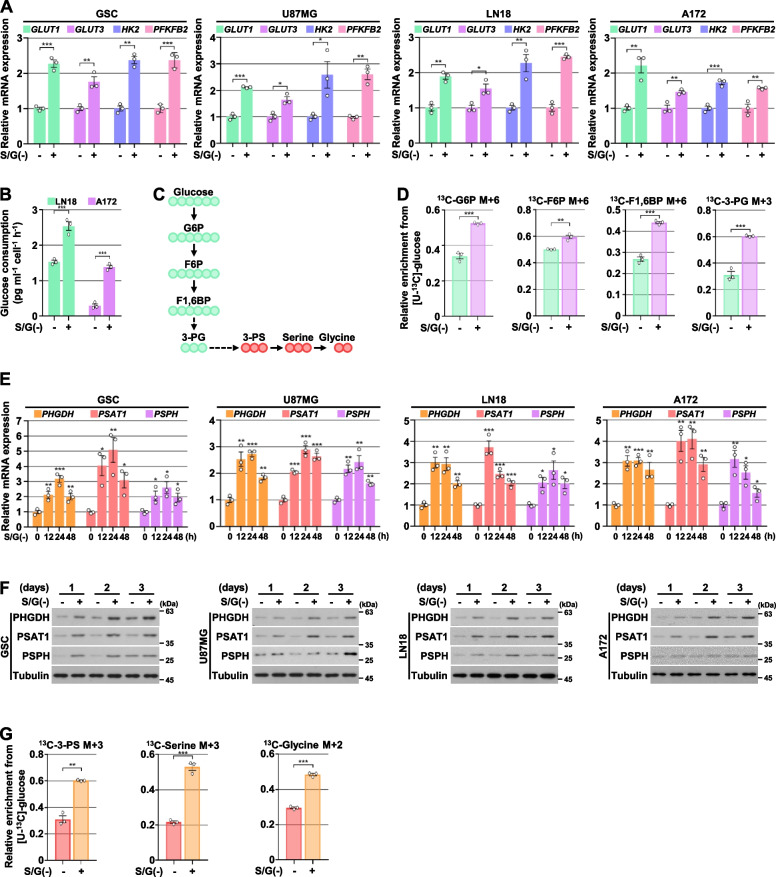
Serine/glycine deprivation induces glucose uptake, glycolytic flux, and de novo serine biosynthesis. **A** Indicated cells were cultured with or without S/G(-) media for 24 h. Quantitative real-time PCR analyses were performed with the indicated primers. **B** Indicated cells were cultured with or without S/G(-) media for 48 h. Glucose consumption was measured. **C** Schematic of [U-^13^C_6_]-glucose into glycolysis and serine synthesis pathway. Glucose (M + 6) is converted into 3-PG (M + 3), a three-carbon sugar, through several steps. Serine (M + 3) is synthesized from the glycolytic intermediate 3-PG, and then glycine (M + 2) is generated form the serine. **D** Isotopomer distributions of G6P, F6P, F1,6BP, 3-PG from [U-^13^C_6_]-glucose in U87MG cells cultured with or without S/G(-) media, n = 3 biologically independent samples. **E** and **F** Indicated cells were cultured without S/G(-) media for the indicated periods of time. Quantitative real-time PCR (E) and Immunoblotting (F) analyses were performed with the indicated antibodies and primers, respectively. **G** Isotopomer distributions of 3-PS, serine, and glycine from [U-^13^C_6_]-glucose in U87MG cells cultured with or without S/G(-) media, n = 3 biologically independent samples. The data represent the mean ± s.d. of three independent experiments (**A, B, D, E, G**). *P < 0.05; **P < 0.01; ***P < 0.001, based on the Student’s t-test

Analyses of quantitative real-time PCR confirmed that S/G deprivation significantly induced the mRNA expression levels of *GLUT1*, *GLUT 3*, *HK2*, and *PFKFB2* in glioma stem cells (GSCs) and GBM cells, including U87MG, LN18, and A172 (Fig. 2A). As expected, glucose consumption was increased by S/G deprivation in GBM cells (Fig. 2B). In line with this result, the metabolic flux analysis with [U-^13^C_6_]-glucose showed that S/G deprivation significantly induced the metabolic flux to glycolysis, including M + 6 glucose-6-phosphate (G6P), M + 6 fructose-6-phosphate (F6P), M + 6 fructose-1,6-bisphosphate (F1,6BP), and M + 3 3-phosphoglycerate (3-PG) in U87MG cells (Fig. 2C and D). We further confirmed the expression of SSP enzymes using analyses of real-time PCR and immunoblotting and showed that S/G deprivation largely induced mRNA and protein expression levels of all three SSP enzymes (Fig. 2E and F), which was reversed by the addition of serine or/and glycine into the S/G-deprived GBM cells (Fig. S2A of the SI). The inhibition of serine or/and glycine uptake by treatment with 4-fluoro-L-2-phenylglycine (a D-serine transport inhibitor) or sarcosine (glycine transporter I inhibitor) also resulted in the induced protein expression levels of all three SSP enzymes in GBM cells cultured in S/G-enriched medium (Fig. S2B of the SI). As expected, the intracellular amounts of ^13^C-labeled M + 3 3-PS, M + 3 serine, and M + 2 glycine were increased by S/G deprivation (Fig. 2G), which were evidenced by [U-^13^C_6_]-glucose-mediated metabolic tracing. Taken together, these results indicate that S/G deficiency induces the expression of genes associated with glycolytic metabolism and SSP and promotes glucose-derived de novo serine and glycine biosynthesis in GBM cells.

### Serine synthesis pathway genes are overexpressed in gliomas and required for brain tumor growth

**Fig. 3:**
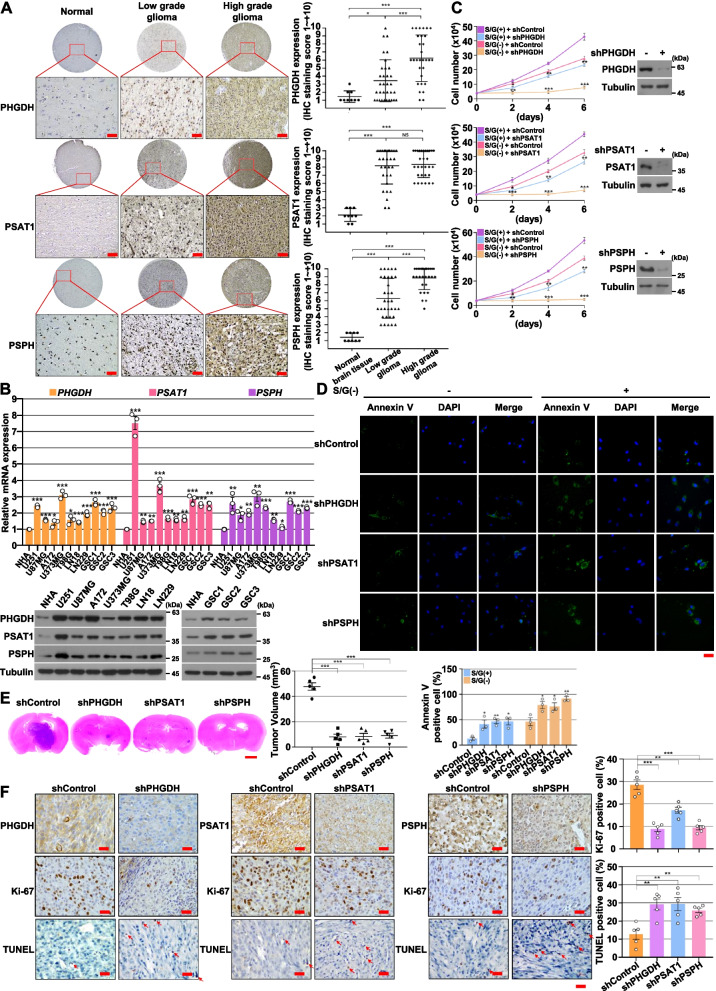
Serine synthesis pathway genes are overexpressed in gliomas and required for brain tumor growth. **A** Microarrays of human gliomas and normal brain tissue were immunostained with the indicated antibodies. Representative images are shown (left panel). Data represent the mean ± s.d. (right panel). Scale bar, 100 μm. NS, not significant. **B** The mRNA and protein expression levels of PHGDH, PSAT1, and PSPH in NHA, GSCs and the indicated GBM cells were determined by quantitative real-time PCR (top panel) and immunoblotting (bottom panel) analyses with the indicated primers and antibodies, respectively. **C** U87MG cells with or without the depletion of SSP genes were cultured with or without S/G(-) media for the indicated periods of time, and harvested for cell counting. **D** U87MG cells with or without the depletion of SSP genes were cultured with or without S/G(-) media for 4 d, and stained with Annexin V. Representative staining (top panel) and quantification of the staining (bottom panel) are shown. Scale bar, 20 μm. **E** A total of 5 × 10^5^ control GSCs or SSP-depleted GSCs were intracranially injected into athymic nude mice. After 21 d, the mice were euthanized and examined for tumor growth. Hematoxylin-and-eosin–stained coronal brain sections show representative tumor xenografts (left panel). Tumor volumes were measured using the length (*a*) and width (*b*) and calculated using the equation *V* = *ab*^*2*^*/2*. Data represent the mean ± s.d. of 5 mice (right panel). Note that the scores of some samples overlap. Scale bar, 2 mm. **F** IHC analyses of the tumor tissues were performed with anti-PHGDH, anti-PSAT1, anti-PSPH, and anti-Ki-67 antibodies and TUNEL. Representative staining (left panel) and quantification of the staining (right panel) are shown. Scale bar, 100 μm. The data represent the mean ± s.d. of three independent experiments (**A-D, F**). *P < 0.05; **P < 0.01; ***P < 0.001, based on the Student’s t-test

We next evaluated the expression levels of SSP enzymes in different grades of human glioma specimens and normal human brain tissue samples from the same patients. The results of immunohistochemical (IHC) staining showed that PHGDH, PSAT1, and PSPH expression levels in both low-grade gliomas and high-grade gliomas (GBM) are much higher than those in normal human brain tissue (Fig. 3A). In addition, analyses of The Gene Expression Omnibus (GEO) database (GSE4290; n = 180) showed that PSAT1 and PSPH genes are upregulated in human gliomas tissues, compared to normal tissues (Fig. S3A of the SI). In line with these findings, quantitative real-time PCR and immunoblotting analyses revealed that human GBM cells and GSCs exhibited higher mRNA and protein expression levels of PHGDH, PSAT1, and PSPH than human normal astrocytes (NHA) (Fig. 3B).

To determine the role of SSP genes in the proliferation and survival of GBM and brain tumor growth, we depleted SSP genes using validated corresponding short hairpin RNA (shRNA) [38] in GBM cells. Depletion of SPP genes resulted in reduced cell proliferation (Fig. 3C and Fig. S3B of the SI) and induced cell apoptosis in both basal and S/G-deprived conditions (Fig. 3D and Fig. S3C of the SI). Of importance, those effects were more provoked in extracellular S/G-deficient conditions. The SPP genes deficiency-reduced cell proliferation was completely rescued by the ectopic expression of SSP genes (*PHGDH*, *PSAT1*, or *PSPH*) in S/G-deprived conditions (Fig. S3D of the SI). Consistent with these results, a reduction in the expression of PHGDH, PSAT1, or PSPH inhibited the growth of brain tumors derived from intracranially injected GSCs (XO6) [25, 26] in athymic nude mice (Fig. 3E), which was accompanied by the inhibition of cell proliferation, as evidenced by the intensity of Ki-67 expression, and induction of apoptotic cells, as detected by the terminal deoxynucleotidyl transferase dUTP nick end labeling (TUNEL) assay (Fig. 3F). Taken together, these results indicate that SSP genes are highly expressed in human gliomas and play important roles in brain tumorigenesis.

### HIF-1α induces SSP gene expression in response to serine/glycine deprivation

**Fig. 4:**
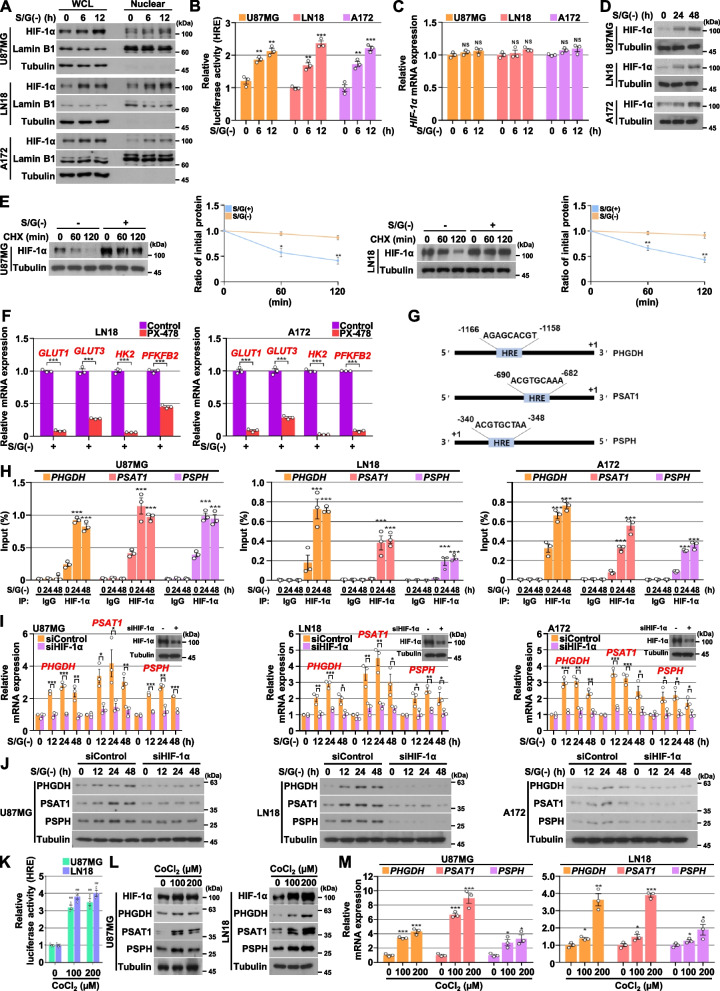
HIF-1α induces SSP gene expression in response to serine/glycine deprivation. **A** Indicated cells were cultured with or without S/G(-) media for the indicated periods of time. Whole cell lysates (WCL) and nuclear fractions were prepared, and immunoblotting analyses were then performed with the indicated antibodies. **B** HRE luciferase activities were measured in the indicated cells culturing with or without S/G(-) media for the indicated periods of time. **C** Indicated cells were cultured with or without S/G(-) media for the indicated periods of time. Quantitative real-time PCR analyses were performed with the indicated primers. **D** Indicated cells were cultured with or without S/G(-) media for the indicated periods of time. Immunoblotting analyses were performed with the indicated antibodies. **E** Indicated cells were cultured with or without S/G(-) media for 24 h, and then treated with CHX (100 μg⋅mL^−1^) for the indicated periods of time. Immunoblotting analyses were performed with the indicated antibodies. Quantification of HIF-1α levels relative to tubulin is shown. Data represent the mean ± s.d. of three independent experiments. **F** Indicated cells were cultured with S/G(-) media in the presence or absence of PX-478 (10 μM) for 24 h. Quantitative real-time PCR analyses were performed with the indicated primers. **G** Schematics of the putative HIF-1α binding site on the *PHGDH*, *PSAT1*, and *PSPH* promoter regions, respectively. **H** Indicated cells were cultured with or without S/G(-) media for the indicated periods of time. ChIP assays were performed with anti-HIF-1α antibody, and quantitative real-time PCR analyses were performed with primers against the *PHGDH*, *PSAT1*, and *PSPH* promoters. **I** and **J** Indicated cells with or without the depletion of HIF-1α were cultured with or without S/G(-) media for the indicated periods of time. Quantitative real-time PCR (I) and immunoblotting (J) analyses were performed with the indicated primers and antibodies, respectively. **K** HRE luciferase activities were measured in the indicated cells cultured in complete media with or without CoCl_2_ for 24 h. **L** and **M** Indicated cells were cultured in complete media with or without CoCl_2_ for 24 h. Immunoblotting (L) and quantitative real-time PCR (M) analyses were performed with the indicated antibodies and primers, respectively. The data represent the mean ± s.d. of three independent experiments (**B, C, E, F, H, I, K, M**). *P < 0.05; **P < 0.01; ***P < 0.001, based on the Student’s t-test

It is well known that HIF-1α reprograms glucose metabolism, which is achieved by the HIF-1α-dependent upregulation of genes encoding glucose transporters and enzymes of the glycolytic pathway [39]. Given that S/G deprivation induced the expression of known HIF-1α-dependent genes (i.e., *GLUT1*, *GLUT 3*, *HK2,* and *PFKFB2)* [39] (Figs. 1 and 2), we hypothesized that HIF-1α transcriptional activity is induced under the S/G-limiting conditions. As expected, S/G deprivation induced HIF-1α nuclear accumulation (Fig. 4A) and transcriptional activity (Fig. 4B), accompanied by the upregulation of HIF-1α protein expression (Fig. 4A) with unaltered mRNA expression (Fig. 4C). HIF-1α upregulation was sustained in (24 or 48) h period (Fig. 4D) in GBM cells. In addition, the HIF-1α protein stabilities were enhanced in S/G-deprived conditions, compared with those in S/G-enriched conditions (Fig. 4E). Next, to determine whether HIF-1α could regulate S/G deprivation-induced genes involved in glucose transporters and glycolytic pathway, we treated with HIF-1α inhibitor PX-478, which successfully suppressed its protein expression (Fig. S4A of the SI) and transcriptional activity (Fig. S4B of the SI), and showed that HIF-1α inhibition resulted in the decreased mRNA expression levels of *GLUT1*, *GLUT 3*, *HK2*, and *PFKFB2* induced by S/G deprivation in GBM cells (Fig. 4F).

To investigate whether HIF-1α directly regulates the transcriptional expression of SSP genes in response to S/G deprivation, we analyzed promoters of the SSP genes and found that hypoxia responsive element (HRE), a HIF-1-binding site, exists in all promoter regions of *PHGDH*, *PSAT1*, and *PSPH*, respectively (Fig. 4G). A chromatin immunoprecipitation (ChIP) assay with an anti-HIF-1α antibody showed that S/G deprivation increased the binding of HIF-1α to the promoter regions of *PHGDH*, *PSAT1*, and *PSPH* in GBM cells (Fig. 4H). As expected, depletion of HIF-1α expression using siRNA, shRNA, or PX-478 resulted in impaired S/G deprivation-induced mRNA and protein expression levels of PHGDH, PSAT1, and PSPH in GBM cells (Fig. 4I and J, and Fig.S4C and D of the SI). In contrast, an increase in HIF-1α transcriptional activity (Fig. 4K) and its protein expression (Fig. 4L) by treatment with CoCl_2_, a chemical inducer of HIF-1, led to upregulated expression of all SSP genes at their mRNA (Fig. 4M) and protein (Fig. 4L) levels in GBM cells cultured in S/G-enriched medium. Collectively, these results suggest that HIF-1α binds to the promoter regions, and enhances the transcriptional expression of SSP genes in response to S/G deprivation.

### AMPK activation is required for the HIF-1α-induced SSP gene expression in response to serine/glycine deprivation

**Fig. 5:**
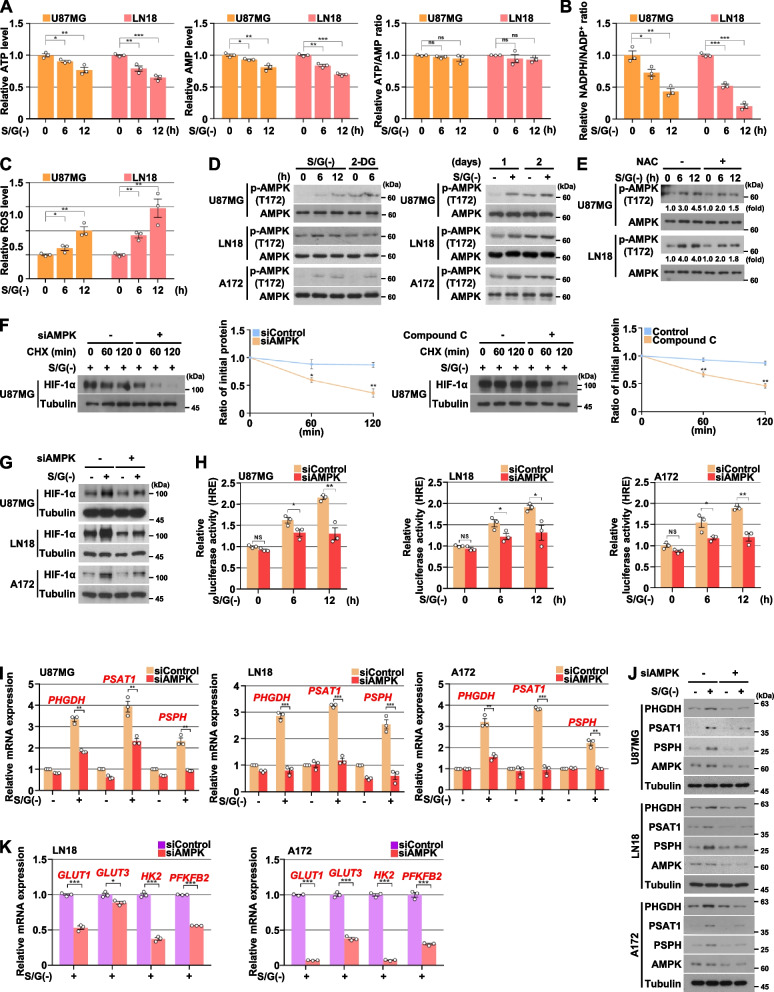
AMPK activation is required for the HIF-1α-induced SSP gene expression in response to serine/glycine deprivation. **A-C** Indicated cells were cultured with or without S/G(-) media for the indicated periods of time. Intracellular ATP and AMP levels (A), NADPH and NADP^+^ levels (B), and ROS levels (C) were measured. NS, not significant. **D** Indicated cells were cultured with or without S/G(-) media in the presence or absence of 2-DG (10 mM) for the indicated periods of time. Immunoblotting analyses were performed with the indicated antibodies. **E** Indicated cells were cultured with or without S/G(-) media for the indicated periods of time in the presence or absence of NAC (2 mM). Immunoblotting analyses were performed with the indicated antibodies. **F** Indicated cells were cultured with S/G(-) media for 24 h in the presence or absence of compound C (5 μM), and then treated with CHX (100 μg⋅mL^−1^) for the indicated periods of time. Immunoblotting analyses were performed with the indicated antibodies. Quantification of HIF-1α levels relative to tubulin is shown. Data represent the mean ± s.d. of three independent experiments. **G** Indicated cells with or without the depletion of AMPK were cultured with or without S/G(-) media for 24 h. Immunoblotting analyses were performed with the indicated antibodies. **H** HRE luciferase activities were measured in the indicated cells with or without the depletion of AMPK culturing with or without S/G(-) media for the indicated periods of time. NS, not significant. **I** and **J** Indicated cells with or without the depletion of AMPK were cultured with or without S/G(-) media for 24 h. Quantitative real-time PCR (I) and immunoblotting (J) analyses were performed with the indicated primers and antibodies, respectively. **K** Indicated cells were cultured with S/G(-) media in the presence or absence of compound C (10 μM) for 24 h. Quantitative real-time PCR analyses were performed with the indicated primers. The data represent the mean ± s.d. of three independent experiments (**A-C, F, H, I, K**). *P < 0.05; **P < 0.01; ***P < 0.001, based on the Student’s t-test

Catabolism of serine and glycine provides one-carbon units for the one-carbon metabolism, which is required for de novo purine synthesis and for a redox balance via NADPH production-mediated reactive oxygen species (ROS) detoxification [17, 18]. We observed that S/G starvation caused decreased adenosine triphosphate (ATP) levels (Fig. 5A, left panel) and NADPH/NADP^+^ ratio (Fig. 5B), and increased ROS levels (Fig. 5C). AMP-activated protein kinase (AMPK), the key metabolic sensor and regulator, could respond to the changed cellular ATP or ROS levels [40–42]. Thus, we were prompted to check AMPK activity under S/G deprivation conditions. We showed that S/G deprivation caused an immediate increase in AMPK phosphorylation at Thr (T)172, which was sustained even in long-term cultures in GBM cells (Fig. 5D). AMPK T172 phosphorylation was also increased in serine and/or glycine transporter inhibitor-treated GBM cells (Fig. S5A of the SI). As S/G starvation also caused lower levels of adenosine monophosphate (AMP) (Fig. 5A, middle panel) resulting in unchanged ATP/AMP ratio (Fig. 5A, right panel), we surmised that the AMPK activation might be ROS-dependent. As expected, the addition of ROS scavenger NAC (N-Acetyl-L-Cysteine) to the culture medium reversed the S/G deprivation-increased AMPK T172 phosphorylation (Fig. 5E and Fig. S5C of the SI), suggesting the role of ROS in AMPK activation under S/G starvation conditions.

AMPK has been found to be involved in the regulation of HIF-1α expression and transactivation [43–45].We next determined whether AMPK activation is required for S/G deprivation-induced HIF-1α expression and transactivation. A reduction of AMPK as a result of AMPKα1 siRNA expression or treatment of a selective AMPK inhibitor compound C significantly diminished S/G deprivation-induced HIF-1α stability (Fig. 5F) and expression (Fig. 5G and Fig. S5B of the SI) and its transcriptional activity (Fig. 5H and Fig. S5D of the SI) in GBM cells. As expected, the depletion of AMPK expression or compound C treatment resulted in impaired S/G deprivation-induced mRNA and protein expression levels of PHGDH, PSAT1, and PSPH (Fig. 5I and J, and Fig. S5E and F of the SI), as well as mRNA expression levels of *GLUT1*, *GLUT 3*, *HK2*, and *PFKFB2* (Fig. 5K) in GBM cells. These results indicate that S/G deprivation induces gene expression of glycolytic metabolism and SSP in a manner dependent on AMPK activation-mediated HIF-1α transactivation.

### AMPK-HIF-1α signaling promotes de novo serine biosynthesis, proliferation, and survival of GBM cells upon serine/glycine deprivation

**Fig. 6:**
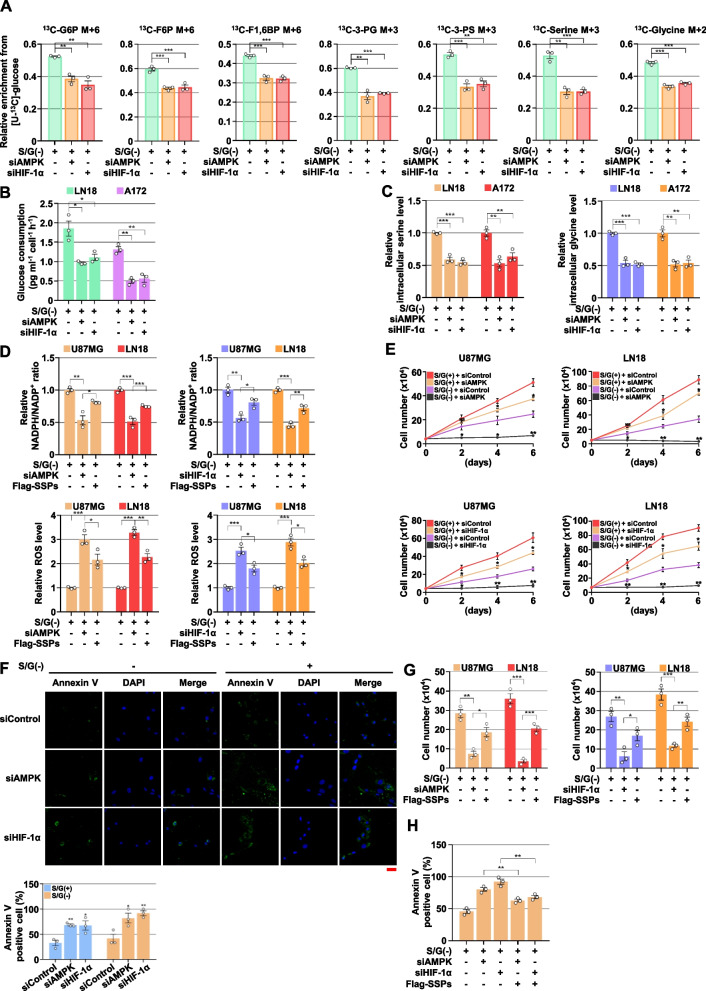
AMPK-HIF-1α signaling promotes de novo serine biosynthesis, proliferation, and survival of GBM cells upon serine/glycine deprivation. **A** Isotopomer distributions of G6P, F6P, F1,6BP, 3-PG, 3-PS, serine, and glycine from [U-^13^C_6_]-glucose in U87MG cells cultured with S/G(-) media with or without the depletion of AMPK or HIF-1α, *n *= 3 biologically independent samples. **B** and **C** Indicated cells with or without the depletion of AMPK or HIF-1α were cultured with S/G(-) media for 2 d (to measure glucose consumption), or 5 d (to measure intracellular serine/glycine levels). Glucose consumption (B) and intracellular serine/glycine levels (C) were measured. **D** Indicated cells with or without the depletion of AMPK or HIF-1α and with or without the reconstituted expression of SSP genes were cultured with S/G(-) media for 5 d. Intracellular NADPH and NADP^+^ levels (top panel) and ROS levels (bottom panel) were measured. **E** Indicated cells with or without the depletion of AMPK or HIF-1α were cultured with or without S/G(-) media for the indicated periods of time, and harvested for cell counting. NS, not significant. **F** U87MG cells with or without the depletion of AMPK or HIF-1α were cultured with or without S/G(-) media for 4 d, and stained with Annexin V. Representative staining (top panel) and quantification of the staining (bottom panel) are shown. Scale bar, 20 μm. **G** and **H** Indicated cells with or without the depletion of AMPK or HIF-1α, and with or without the reconstituted expression of SSP genes, were cultured with S/G(-) media for 4 d (for Annexin V staining) or 6 d (for cell counting). The data represent the mean ± s.d. of three independent experiments (**A-H**). *P < 0.05; **P < 0.01; ***P < 0.001, based on the Student’s t-test or one-way ANOVA with Tukey’s post hoc test

Because the AMPK-HIF-1α axis regulates metabolic genes associated with glucose uptake, glycolytic process, and SSP under the limited S/G, we next investigated the roles of AMPK and HIF-1α in glucose-derived de novo serine and glycine biosynthesis. Metabolic flux analysis using [U-^13^C_6_]-glucose revealed that the depletion of AMPK or HIF-1α significantly decreased the S/G deprivation-induced metabolic flux to serine biosynthesis pathway, including ^13^C-labelled G6P, F6P, F1,6BP, 3-PG, 3-PS, serine, and glycine (Fig. 6A). In line with these results, the inhibition of AMPK or HIF-1α resulted in reduced S/G deprivation-induced glucose consumption (Fig. 6B) and intracellular serine and glycine levels (Fig. 6C and Fig. S6A of the SI). These results demonstrate that AMPK-HIF-1α signaling promotes the flux of glucose-derived de novo serine and glycine biosynthesis in S/G-deficient conditions. Given that depletion of SSP genes caused decreased levels of intracellular serine and glycine (Fig. S6B of the SI) and NADPH/NADP^+^ ratio (Fig. S6C of the SI), and increased ROS levels (Fig. S6D of the SI) under S/G-deprived conditions, we postulated that AMPK-HIF-1α signaling maintains one-carbon metabolism-mediated redox homeostasis via regulation of SSP activation. As expected, the S/G deprivation-reduced levels of NADPH/NADP^+^ ratio and -induced levels of ROS were exacerbated by the inhibition of AMPK or HIF-1α, which were significantly reversed by the ectopic expression of SSP genes (*PHGDH*, *PSAT1*, and *PSPH*) (Fig. 6D and Fig. S6E of the SI). Next, we further investigated the effect of AMPK and HIF-1α on cell proliferation and survival under limited S/G conditions. Consistent with the roles of SSP genes on proliferation and survival (Fig. 3), the inhibition of AMPK or HIF-1α attenuated cell proliferation (Fig. 6E) and accelerated cell apoptosis (Fig. 6F) both in basal, and with more obvious effects, in S/G-deprived conditions. Of note, the inhibited proliferation and increased apoptosis by the depletion of AMPK or HIF-1α were partially restored by the ectopic expression of SSP genes in S/G-free conditions (Fig. 6G and H). In addition, the inhibition of glycolysis using its inhibitor 2-deoxy-D-glucose (2-DG) treatment decreased the proliferation of GBM cells more dramatically under S/G-deprived conditions than under normal condition (Fig. S6F of the SI). Taken together, these results suggest that GBM cells have a glucose metabolic dependency, and that AMPK-HIF-1α signaling significantly contributes to the glucose-derived de novo serine biosynthesis, redox homeostasis, proliferation, and survival of GBM cells under the limited serine and glycine conditions.

### The expression levels of SSP enzymes are positively correlated with levels of AMPK T172 phosphorylation and HIF-1α expression in human GBM specimens and poor GBM patient prognosis

**Fig. 7:**
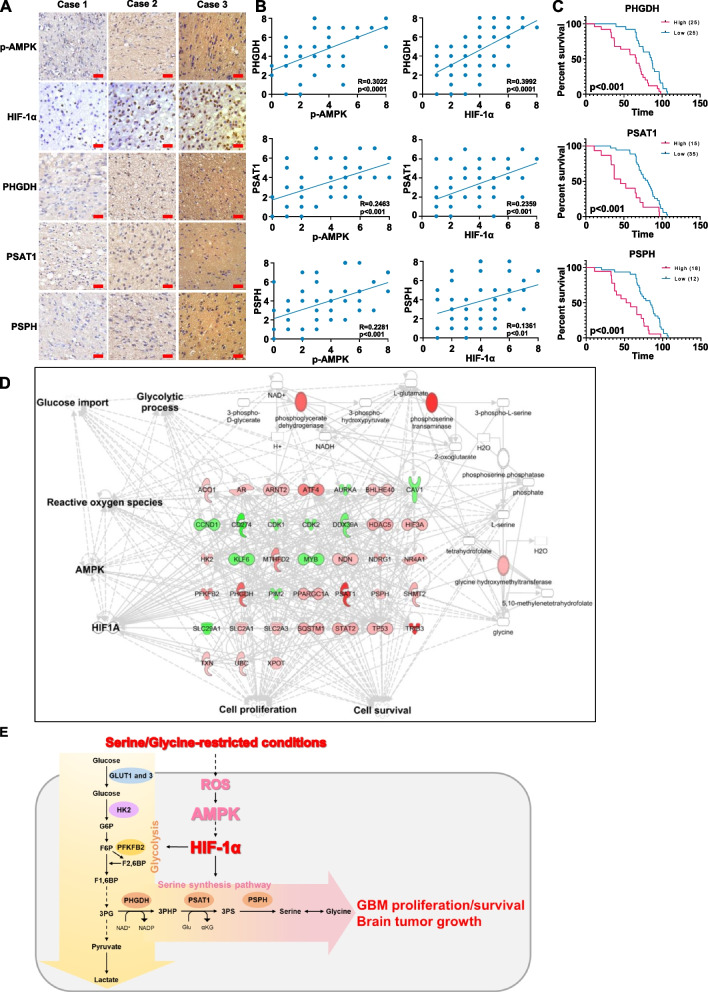
The expression levels of SSP enzymes are positively correlated with levels of AMPK T172 phosphorylation and HIF-1α expression in human GBM specimens and poor GBM patient prognosis. **A** IHC staining of human GBM specimens was performed with the indicated antibodies (*n* = 50). Representative images from the staining of four different specimens are shown. Scale bar, 100 μm. **B** The IHC stains were scored, and correlation analyses were performed. The Pearson correlation test was used. Note that the scores of some samples overlapped. **C** Kaplan–Meier analyses for GBM patients with high or low level of SSP. **D** Network with DEGs, biological functions, glycolytic metabolism, and SSP using IPA in S/G-deprived U87MG cells, compared to control. The analysis involved a fold change cut-off value of ± 1.3. Red and green colors indicate genes that were upregulated and downregulated, respectively, compared to the control. Reactive oxygen species (ROS), AMPK, and HIF-1α are added for analysis of the relationships among the genes for the biological functions, glycolytic metabolism, and SSP. Figure S1A of the SI provides the details of shape, which originate from Ingenuity Systems (http://www.ingenuity.com). **E** Schematic of the regulation of Brain Tumor Serine metabolism (BTS); AMPK-HIF-1α signaling-enhanced glucose-derived de novo serine biosynthesis to promote the proliferation and survival of GBM and brain tumor growth under the brain microenvironment of limited serine/glycine

To determine the clinical significance of AMPK-HIF-1α signaling-mediated SSP activation, we analyzed 50 primary human GBM specimens. The levels of AMPK pT172 and HIF-1α expression were positively correlated with the expression levels of PHGDH, PSAT1, and PSPH (Fig. 7A). Quantification of staining showed that these correlations were significant (Fig. 7B). We next compared the survival duration of 50 patients, all of whom had undergone standard adjuvant radiotherapy after the surgical resection of GBM, followed by treatment with an alkylating agent (temozolomide, in most cases). The median survival durations were 86.6, 81, and 84.05 weeks for patients whose tumors had low levels of PHGDH, PSAT1, and PSPH, respectively, and 66.2, 45.6, and 55.2 weeks for those whose tumors had high levels of PHGDH, PSAT1, and PSPH, respectively (Fig. 7C). These results indicate that AMPK-HIF-1α signaling-mediated SSP activation plays an important role in the clinical behavior of human GBM and reveal a significant correlation between the levels of AMPK pT172 and HIF-1α expression, the expression levels of PHGDH, PSAT1, and PSPH and the clinical aggressiveness of GBM.

## Discussion

Cancer cells acquire distinct metabolic reprogramming to survive diverse stress conditions and to satisfy the anabolic demands of rapid proliferation for tumor growth and metastasis [6–8]. Thus, during tumor development, cancer metabolic reprogramming-based flexibility is commonly observed. Here, we investigated the mechanisms of how GBM cells achieve their distinct rewired metabolic features to contribute to survival and proliferation within the brain microenvironment. We revealed that GBM exhibited metabolic alterations in enforced glucose-derived de novo serine biosynthesis to promote brain tumor growth under the brain microenvironment of limited serine and glycine. ROS-mediated AMPK activation induced HIF-1α expression and transactivation to simultaneously increase the transcriptional expression of the three SSP genes as well as the expression of genes for glucose transporters and glycolytic process, thereby enhancing glucose uptake, glycolytic flux, and de novo serine and glycine biosynthesis to promote the survival and proliferation of GBM cells and brain tumor growth under the brain microenvironment of restricted serine/glycine (Fig. 7D and E). Our findings highlight that under the limited serine and glycine conditions, GBM cells have an increased dependency on glucose-derived carbon for the biosynthesis of serine and glycine in an AMPK/HIF-1α activation-dependent manner.

Serine catabolism is essential to produce one-carbon units for the one-carbon metabolism needed for cancer cell survival and proliferation [17, 18]; thus, recent attention has focused on the role and regulation of serine metabolism in supporting tumor development [9–15, 21, 46]. Although cancer cells can uptake serine from the extracellular environment, many studies have shown that de novo serine synthesis is activated by several factors in many types of cancer [14, 19, 47–49] and thus most cancer cells can adapt to and become resistant to the deficiency of exogenous serine by upregulating the SSP flux. In particular, the growing GBM cells rely on the de novo-synthesized serine to survive and proliferate in the brain microenvironment of restricted serine/glycine. Indeed, we found that the levels of the three SSP enzymes were overexpressed in human GBM specimens and GBM cells, and SSP activation was upregulated in response to the deprivation of serine/glycine. In accordance, our in vitro and in vivo experiments clearly showed that the genetic downregulation of SSP genes suppressed GBM survival and proliferation and brain tumor growth within the limited serine/glycine conditions, implying that serine biosynthesis is a crucial metabolic process for GBM growth. In line with our results, Ngo et al. reported that brain-metastasized tumors exhibited an increase in the expression of PHGDH and PSAT1 genes and serine synthesis relative to extracranial tumors, thereby potentiating brain metastasis [21]. Our and other studies indicate that de novo serine biosynthesis is hyperactivated in the brain microenvironment of restricted serine/glycine, which plays roles in both brain tumor growth and brain metastasis. Thus, in future studies, attempting therapeutic targeting of SSP enzymes in tumors growing in the brain microenvironment will be worth exploring.

It is well known that glucose is the major source for the biosynthesis of serine and glycine. Nevertheless, it is quite unclear how glucose-derived serine biosynthesis is comprehensively coordinated to support cellular survival and proliferation under stressed conditions, including serine/glycine deficiency. HIF-1α plays a critical role in glycolytic metabolism through upregulating glycolysis-related enzymes in cancer cells [39]. A highly active glucose metabolism [50] and overexpression of HIF-1α [51] have been observed in GBM, and high levels of HIF-1α are associated with advanced cancer progression and poor clinical outcomes in GBM [51, 52]. Thus, HIF-1α can contribute to supplying glucose-derived precursor 3-phosphoglycerate for de novo serine biosynthesis in GBM cells. Our transcriptomic data showed that the genes for SSP, glucose transporters, and glycolytic process are highly upregulated in response to serine/glycine deprivation. We demonstrate here that activated transcription factor HIF-1α simultaneously controls the expression of GLUT1, GLUT3, HK2, PFKFB2, PHGDH, PSAT1, and PSPH, and then enhanced glucose uptake, glycolytic flux, and de novo serine biosynthesis under the limited serine/glycine conditions. These findings highlight that HIF-1α acts as a master metabolic coordinator of glucose-derived serine biosynthesis in GBM cells.

In the present study, we found that serine/glycine-deficient conditions enhance HIF-1α stability and transactivation in GBM cells. It has been well reported that both the protein expression and the activity of HIF-1α are regulated by O_2_-dependent pathways [39]. However, recent studies showed that HIF-1α is subject to O_2_-independent regulations. Interplays between AMPK and HIF-1α have been reported that increased AMPK expression and activity are paralleled by the upregulation of HIF-1α in cancer cells [53]. AMPK has been proven to positively regulate HIF-1α protein stability and function. Indeed, the inhibition of AMPK attenuated HIF-1α target genes expression via impairing the expression and nuclear accumulation of HIF-1α in an O_2_-independent pathway manner under hypoxia or low glucose conditions [43–45]. Consistent with the results, serine/glycine deprivation-activated AMPK was required for HIF-1α stability and transactivation in GBM cells (Fig. 5). AMPK signaling was found to be hyperactivated in human GBM specimens compared with normal brain, which supports tumor bioenergetics, growth, and survival in GBM [43], indicating the significance of AMPK activity in GBM. Here, we demonstrate that AMPK activation in human GBM cells resulted from increased ROS, not from change in ATP/AMP ratio under serine/glycine-defective brain microenvironmental conditions. Collectively, through comprehensive molecular and pharmacological approaches, we here show a mechanism by which AMPK induces HIF-1α stability and transactivation, resulting in enhanced de novo serine biosynthesis to support GBM proliferation and survival under serine/glycine deprivation conditions.

## Conclusions

Our study strongly demonstrates that GBM exhibits metabolic dependency in glucose-derived de novo serine biosynthesis to overcome serine/glycine-defective brain microenvironmental stress, and our data defines the central role of ROS-AMPK-HIF-1α signaling in regulating the glucose-derived de novo serine biosynthesis to support the proliferation and survival of GBM cells and brain tumor growth under the limited serine and glycine of the brain microenvironment. Thus, targeting these processes could offer therapeutic potential for treating GBM patients.

### Supplementary Information

**Additional file 1: Table S1.** Enriched top 10 gene ontology annotations for biological process in transcriptome of S/G-deprived U87MG cells, compared to control. **Table S2.** Enriched top 10 gene ontology annotations for molecular function in transcriptome of S/G-deprived U87MG cells, compared to control. **Table S3.** Enriched top 10 gene ontology annotations for cellular component in transcriptome of S/G-deprived U87MG cells, compared to control. **Table S4.** Differentially expressed transcriptome of S/G-deprived U87MG cells, compared to control, in trimmed IPA network. **Figure S1.** (related to Figure 1). DEGs network analyses of transcriptome from U87MG cells in response to serine/glycine deprivation. **Figure S2.** (related to Figure 2). Serine/glycine deprivation induces glucose uptake, glycolytic flux, and de novo serine biosynthesis. **Figure S3.** (related to Figure 3). Serine synthesis pathway genes are overexpressed in gliomas and required for brain tumor growth. **Figure S4.** (related to Figure 4). HIF-1α induces SSP gene expression in response to 3 serine/glycine deprivation. **Figure S5.** (related to Figure 5). AMPK activation is required for the HIF-1α-induced SSP gene expression in response to serine/glycine deprivation. **Figure S6.** (related to Figure 6). AMPK-HIF-1α signaling promotes de novo serine biosynthesis, proliferation, and survival of GBM cells upon serine/glycine deprivation. **Figure S7.** Full uncut blots are presented for the representative western blots featured in the present study.

## Data Availability

All data generated or analyzed during this study are included in this published article. RNA-sequencing and quantification data are available in the gene expression omnibus (GEO) repository of National Center for Biotechnology Information (NCBI) under the accession number GSE235422.

## Abbreviations

AMP: Adenosine monophosphate
AMPK: AMP-activated protein kinase
ATP: Adenosine triphosphate
CHX: Cycloheximide
ChIP: Chromatin immunoprecipitation
CoCl_2_: Cobalt (II) chloride
CSF: Cerebrospinal fluid
CT: Cycle threshold
DAB: 3,3’-Diaminobenzidine
DEGs: Differentially expressed genes
DMEM: Dulbecco’s modified Eagle’s medium
F6P: Fructose-6-phosphate
F1,6BP: Fructose-1,6-bisphosphate
GBM: Glioblastoma
GSCs: Glioma stem cells
GEO: Gene Expression Omnibus
GLUT: Glucose transporter
GO: Gene ontology
G6P: Glucose-6-phosphate
HIF: Hypoxia-inducible factor
HK2: Hexokinase 2
HRE: Hypoxia response element
IHC: Immuohistochemistry
IPA: Ingenuity Pathway Analysis
ISF: Interstitial fluid
NAC: N-Acetyl-L-Cysteine
NADPH/NADP: Nicotinamide adenine dinucleotide phosphate
NHA: Normal human astrocyte
PAGE: Polyacrylamide gel electrophoresis
PCR: Polymerase chain reaction
PFKFB2: 6-Phosphofructo-2-kinase/fructose-2,6-biphosphatase 2
PHGDH: Phosphoglycerate dehydrogenase
PSAT1: Phosphoserine aminotransferase 1
PSPH: Phosphoserine phosphatase
RIPA: Radioimmunoprecipitation assay
ROS: Reactive oxygen species
RT: Room temperature
SDS: Sodium dodecyl sulfate
SHMT: Serine hydroxymethyltransferase
S/G: Serine/glycine
SSP: Serine synthesis pathway
TUNEL: Terminal deoxynucleotidyl transferase dUTP nick end labeling
2-DG: 2-Deoxy-D-glucose
3-PG: 3-Phosphoglycerate
3-PHP: 3-Phosphohydroxypyruvate
3-PS: 3-Phosphoserine
