# Supplementary material for: AMPK-HIF-1α signaling enhances glucose-derived de novo serine biosynthesis to promote glioblastoma growth
Source: J Exp Clin Cancer Res. 2023 Dec 15;42:340. doi: 10.1186/s13046-023-02927-3 (PMC10722853; doi:10.1186/s13046-023-02927-3)

Table S1. Enriched top 10 gene ontology annotations for biological process in transcriptome of S/G-deprived U87MG cells, compared to control

Biological processes	-log(p-value)
cellular response to starvation (GO:0009267)	6.40
regulation of autophagy (GO:0010506)	5.51
positive regulation of apoptotic process (GO:0043065)	5.13
regulation of apoptotic process (GO:0042981)	5.10
neutral amino acid transport (GO:0015804)	4.83
cellular response to amino acid starvation (GO:0034198)	4.70
serine family amino acid biosynthetic process (GO:0009070)	4.62
response to amino acid starvation (GO:1990928)	4.61
autophagy of mitochondrion (GO:0000422)	4.56
glycolipid transport (GO:0046836)	4.50

Table S2. Enriched top 10 gene ontology annotations for molecular function in transcriptome of S/G-deprived U87MG cells, compared to control

Molecular functions	-log(p-value)
neutral amino acid transmembrane transporter activity (GO:0015175)	5.81
collagen receptor activity (GO:0038064)	4.50
microtubule binding (GO:0008017)	4.37
insulin-like growth factor II binding (GO:0031995)	3.99
insulin-like growth factor binding (GO:0005520)	3.81
L-amino acid transmembrane transporter activity (GO:0015179)	3.76
L-glutamine transmembrane transporter activity (GO:0015186)	3.59
L-leucine transmembrane transporter activity (GO:0015190)	3.53
insulin-like growth factor I binding (GO:0031994)	3.30
L-aspartate transmembrane transporter activity (GO:0015183)	3.27

Table S3. Enriched top 10 gene ontology annotations for cellular component in transcriptome of S/G-deprived U87MG cells, compared to control

Cellular components	-log(p-value)
lysosome (GO:0005764)	8.90
lysosomal lumen (GO:0043202)	7.77
lytic vacuole (GO:0000323)	7.00
vacuolar lumen (GO:0005775)	6.19
microtubule (GO:0005874)	4.92
secondary lysosome (GO:0005767)	4.56
autolysosome (GO:0044754)	4.11
autophagosome (GO:0005776)	4.03
lytic vacuole membrane (GO:0098852)	3.93
lysosomal membrane (GO:0005765)	3.79

Table S4. Differentially expressed transcriptome of S/G-deprived U87MG cells, compared to control, in trimmed IPA network

Entrez gene name	Symbol	Entrez Gene ID for human	Location	Signal fold change[a]
aconitase 1	ACO1	48	Cytoplasm	1.30
androgen receptor	AR	367	Nucleus	1.44
aryl hydrocarbon receptor nuclear translocator 2	ARNT2	9915	Nucleus	1.47
activating transcription factor 4	ATF4	468	Nucleus	2.63
aurora kinase A	AURKA	6790	Nucleus	-1.31
basic helix-loop-helix family member e40	BHLHE40	8553	Nucleus	1.33
caveolin 1	CAV1	857	Plasma Membrane	-1.34
cyclin D1	CCND1	595	Nucleus	-1.35
CD274 molecule	CD274	29126	Plasma Membrane	-2.04

cyclin dependent kinase 1	CDK1	983	Nucleus	-1.32
cyclin dependent kinase 2	CDK2	1017	Nucleus	-1.43
DExD-box helicase 39A	DDX39A	10212	Nucleus	-1.42
histone deacetylase 5	HDAC5	10014	Nucleus	1.81
hypoxia inducible factor 3 subunit alpha	HIF3A	64344	Nucleus	1.53
hexokinase 2	HK2	3099	Cytoplasm	1.62
KLF transcription factor 6	KLF6	1316	Nucleus	-1.42
methylenetetrahydrofolate dehydrogenase (NADP+ dependent) 2, methenyltetrahydrofolate cyclohydrolase	MTHFD2	10797	Cytoplasm	2.39
MYB proto-oncogene, transcription factor	MYB	4602	Nucleus	-1.38
necdin, MAGE family member	NDN	4692	Nucleus	1.42
N-myc downstream regulated 1	NDRG1	10397	Nucleus	1.80
nuclear receptor subfamily 4 group A member 1	NR4A1	3164	Nucleus	1.60

6-phosphofructo-2-kinase/fructose-2,6-biphosphatase 2	PFKFB2	5208	Cytoplasm	2.40
phosphoglycerate dehydrogenase	PHGDH	26227	Cytoplasm	3.26
Pim-2 proto-oncogene, serine/threonine kinase	PIM2	11040	Nucleus	-1.40
PPARG coactivator 1 alpha	PPARGC1A	10891	Nucleus	1.71
phosphoserine aminotransferase 1	PSAT1	29968	Cytoplasm	4.92
phosphoserine phosphatase	PSPH	5723	Cytoplasm	1.43
serine hydroxymethyltransferase 2	SHMT2	6472	Cytoplasm	1.69
solute carrier family 29 member 1 (Augustine blood group)	SLC29A1	2030	Plasma Membrane	-1.70
solute carrier family 2 member 1	SLC2A1	6513	Plasma Membrane	1.30
solute carrier family 2	SLC2A3	6515	Plasma	1.34

member 3			Membrane	
sequestosome 1	SQSTM1	8878	Cytoplasm	1.78
signal transducer and activator of transcription 2	STAT2	6773	Nucleus	1.75
tumor protein p53	TP53	7157	Nucleus	1.57
tribbles pseudokinase 3	TRIB3	57761	Nucleus	5.89
thioredoxin	TXN	7295	Cytoplasm	1.36
ubiquitin C	UBC	7316	Cytoplasm	1.31
exportin for tRNA	XPOT	11260	Nucleus	1.41

[a]Normalized signal fold change in S/G-deprived U87MG cells group to corresponding signal of control group

Figure S1 (related to Figure 1). DEGs network analyses of transcriptome from U87MG cells in response to serine/glycine deprivation

(A) Analysis of DEGs network using IPA in S/G-deprived U87MG cells, compared to control. The analysis involved a fold change cut-off value of ±1.3. Red and green colors indicate genes that were upregulated and downregulated, respectively, compared to the control. Details for shape, which originate from Ingenuity Systems (http://www.ingenuity.com).

(B) Analysis of trimmed DEGs network with biological functions, including cell proliferation, cell survival, and glucose metabolism disorder, using IPA in S/G-deprived U87MG cells, compared to control. The analysis involved a fold change cut-off value of ±1.3. Red and green colors indicate genes that were upregulated and downregulated, respectively, compared to the control. Details for shape, which originate from Ingenuity Systems (http://www.ingenuity.com), are provided in Figure S1A.

(C) Analysis of trimmed DEGs network with biological functions, including cell proliferation, cell survival, L-serine biosynthetic process, glycolytic process, and glucose import, using IPA in S/G-deprived U87MG cells, compared to control. The analysis involved a fold change cut-off value of ±1.3. Red and green colors indicate genes that were upregulated and downregulated, respectively, compared to the control. Relationships between L-serine biosynthetic process and genes are highlighted with magenta. Details for shape, which originate from Ingenuity Systems (http://www.ingenuity.com), are provided in Figure S1A.

Figure S2 (related to Figure 2). Serine/glycine deprivation induces glucose uptake, glycolytic flux, and *de novo* serine biosynthesis

(A) Indicated cells were cultured with or without S/G(-) media for 24 h in the presence or

absence of exogenous serine (400 μM) and/or glycine (400 μM). Immunoblotting analyses were performed with the indicated antibodies.

(B) LN18 cells were cultured in complete media for 24 h in the presence or absence of serine transporter inhibitor (4-Fluoro-L-2-phenylglycine; 50 μM) and/or glycine transporter inhibitor (Sarcosine; 50 μM). Immunoblotting analyses were performed with the indicated antibodies.

Figure S3 (related to Figure 3). Serine synthesis pathway genes are overexpressed in gliomas and required for brain tumor growth

(A) GEO dataset (GSE4290) analysis of *PSAT1* and *PSPH* mRNA expression in normal (n = 23) and brain tumor (n = 157) tissues.

(B) LN18 cells with or without the depletion of SSP genes were cultured with or without S/G(-) media for the indicated periods of time, and harvested for cell counting.

(C) LN18 cells with or without the depletion of SSP genes were cultured with or without S/G(-) media for 4 d, and stained with Annexin V. Representative staining (top panel) and quantification of the staining (bottom panel) are shown. Scale bar, 20 μm.

(D) U87MG cells with the depletion of SSP genes, and with or without the reconstituted expression of SSP genes, were cultured with S/G(-) media for the indicated periods of time, and harvested for cell counting.

The data represent the mean ± s.d. of three independent experiments **(B-D)**. *$P < 0.05$; **$P < 0.01$; ***$P < 0.001$, based on the Student's t-test.

Figure S4 (related to Figure 4). HIF-1α induces SSP gene expression in response to

serine/glycine deprivation

(A) Indicated cells were cultured with or without S/G(-) media for 24 h in the presence or absence of PX-478 (10 μM). Western blot analyses were performed with the indicated antibodies.

(B) HRE luciferase activities were measured in the indicated cells cultured with or without S/G(-) media for 24 h in the presence or absence of PX-478 (10 μM).

(C and **D)** Indicated cells with or without the depletion of HIF-1α (left panel) were cultured with or without S/G(-) media for the indicated periods of time in the presence or absence of PX-478 (10 μM). Quantitative real-time PCR (C) and immunoblotting (D) analyses were performed with the indicated primers and antibodies, respectively.

The data represent the mean ± s.d. of three independent experiments **(B, C)**. **P* < 0.05; ***P* < 0.01; ****P* < 0.001, based on the Student's t-test.

Figure S5 (related to Figure 5). AMPK activation is required for the HIF-1α-induced SSP gene expression in response to serine/glycine deprivation

(A) LN18 cells were cultured in complete media for 12 h in the presence or absence of serine transporter inhibitor (4-Fluoro-L-2-phenylglycine; 50 μM) and/or glycine transporter inhibitor (Sarcosine; 50 μM). Immunoblotting analyses were performed with the indicated antibodies.

(B) Indicated cells were cultured with or without S/G(-) media for 24 h in the presence or absence of compound C (10 μM). Immunoblotting analyses were performed with the indicated antibodies.

(C) Indicated cells were cultured with or without S/G(-) media for 12 h in the presence or

absence of NAC (2 mM). Intracellular ROS levels were measured.

(D) HRE luciferase activities were measured in the indicated cells cultured with or without S/G(-) media for the indicated periods of time in the presence or absence of Compound C (10 μM). NS, not significant.

(E and **F)** Indicated cells were cultured with or without S/G(-) media for the indicated periods of time in the presence or absence of compound C (10 μM). Quantitative real-time PCR (E) and immunoblotting (F) analyses were performed with the indicated primers and antibodies, respectively.

The data represent the mean ± s.d. of three independent experiments **(C-E)**. $^{*}P < 0.05$; $^{**}P < 0.01$; $^{***}P < 0.001$, based on the Student's t-test.

Figure S6 (related to Figure 6). AMPK-HIF-1α signaling promotes *de novo* serine biosynthesis, proliferation, and survival of GBM cells upon serine/glycine deprivation

(A) Indicated cells were cultured with S/G(-) media for 5 d in the presence or absence of compound C (10 μM) or PX-478 (5 μM). Intracellular serine and glycine levels were measured.

(B-D) Indicated cells with or without the depletion of SSP gens were cultured with S/G(-) media for 5 d. Intracellular serine and glycine levels (B), NADPH and $NADP^{+}$ levels (C), and ROS levels (D) were measured.

(E) Indicated cells with or without the reconstituted expression of SSP genes were cultured with S/G(-) media for 5 d in the presence or absence of compound C (10 μM) or PX-478 (5 μM). Intracellular NADPH and $NADP^{+}$ levels (top panel), and ROS levels (bottom panel) were measured.

(F) Indicated cells were cultured with or without S/G(-) media for 6 d in the presence or absence of 2-DG (1 mM), and harvested for cell counting.

The data represent the mean ± s.d. of three independent experiments **(A-E)**. *$P < 0.05$; **$P < 0.01$; ***$P < 0.001$, based on the Student’s t-test or one-way ANOVA with Tukey’s *post hoc* test.

Figure S7. Full uncut blots are presented for the representative western blots featured in the present study.

Figure S1

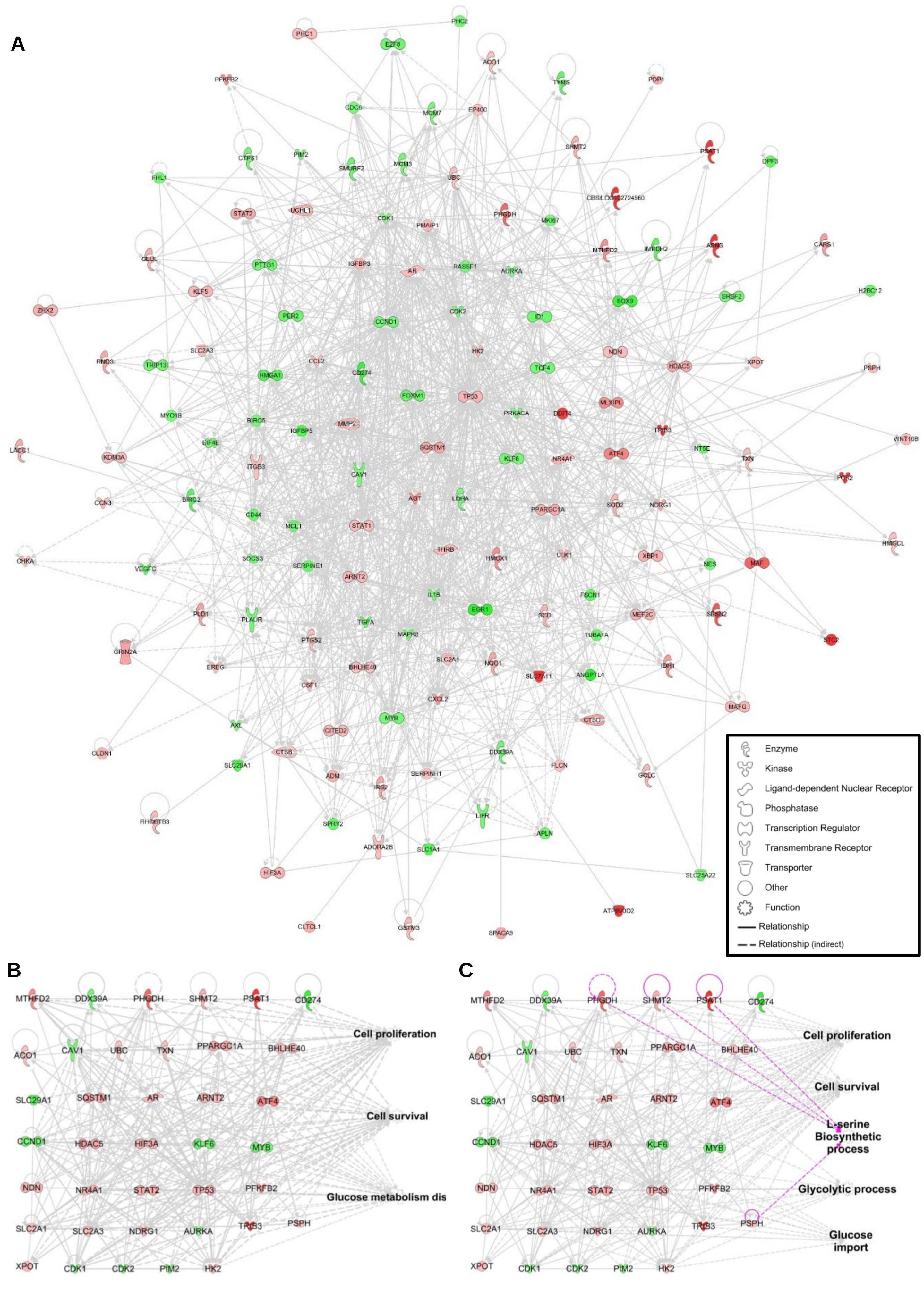
A
PHC2
PHC1
E2F8
ACO1
TYMS
PDP1
PFKFB2
CDC6
MCM7
EP400
SHMT2
PSAT1
DPF3
CTPS1
PIM2
SMURF2
MCM3
UBC
FHL1
CBS/LOC102724560
STAT2
UCHL1
PHGDH
MKI67
CDK1
PMAIP1
MTHFD2
IMPDH2
ASNS
CARS1
GLUL
PTTG1
IGFBP3
AR
RASSF1
AURKA
KLF5
SRSF2
H2BC12
SOX9
ZHX2
PER2
CDK2
ID1
CCND1
SLC2A3
HK2
NDN
RND3
TRIP13
CCL2
XPOT
PSPH
HMGA1
CD274
TCF4
HDAC5
FOXM1
TP53
MLXIPL
MYO1B
PRKACA
DDIT4
BIRC5
MMP2
IGFBP5
TRIB3
WNT10B
LACC1
EIF4E
SQSTM1
NT5E
KDM3A
ATF4
KLF6
NR4A1
TXN
ITGB3
CAV1
POR2
CCN3
BIRC2
AGT
LDHA
PPARGC1A
SOD2
NDRG1
CD44
MCL1
STAT1
HMGCL
CHKA
SOCS3
THRB
HMOX1
ULK1
XBP1
VEGFC
SERPINE1
NES
MAF
ARNT2
IL1B
FSCN1
PLD1
EGR1
PLAUR
SCD
MEF2C
SESN2
TGFA
STC2
GRIN2A
MAPK8
TUBA1A
PTGS2
SLC2A1
NQO1
EREG
BHLHE40
IDH1
SLC7A11
ANGPTL4
CSF1
CXCL2
MAFG
MYB
CTSD
AXL
CITED2
CLDN1
CTSB
DDX39A
FLCN
GCLC
SLC29A1
ADM
SERPINH1
IRS2
RHOBTB3
LIFR
SPRY2
APLN
ADORA2B
SLC1A1
HIF3A
SLC25A22
ATP6V0D2
CLTCL1
GSTM3
SPACA9
Enzyme
Kinase
Ligand-dependent Nuclear Receptor
Phosphatase
Transcription Regulator
Transmembrane Receptor
Transporter
Other
Function
Relationship
Relationship (indirect)
B
MTHFD2
DDX39A
PHGDH
SHMT2
PSAT1
CD274
Cell proliferation
ACO1
CAV1
UBC
TXN
PPARGC1A
BHLHE40
SLC29A1
SQSTM1
AR
ARNT2
ATF4
Cell survival
CCND1
HDAC5
HIF3A
KLF6
MYB
NDN
NR4A1
STAT2
TP53
PFKFB2
Glucose metabolism dis
SLC2A1
SLC2A3
NDRG1
AURKA
TRIB3
PSPH
XPOT
CDK1
CDK2
PIM2
HK2
C
MTHFD2
DDX39A
PHGDH
SHMT2
PSAT1
CD274
Cell proliferation
ACO1
CAV1
UBC
TXN
PPARGC1A
BHLHE40
Cell survival
SLC29A1
SQSTM1
AR
ARNT2
ATF4
L-serine Biosynthetic process
CCND1
HDAC5
HIF3A
KLF6
MYB
NDN
NR4A1
STAT2
TP53
PFKFB2
Glycolytic process
SLC2A1
SLC2A3
NDRG1
AURKA
TRIB3
PSPH
Glucose import
XPOT
CDK1
CDK2
PIM2
HK2

Figure S2

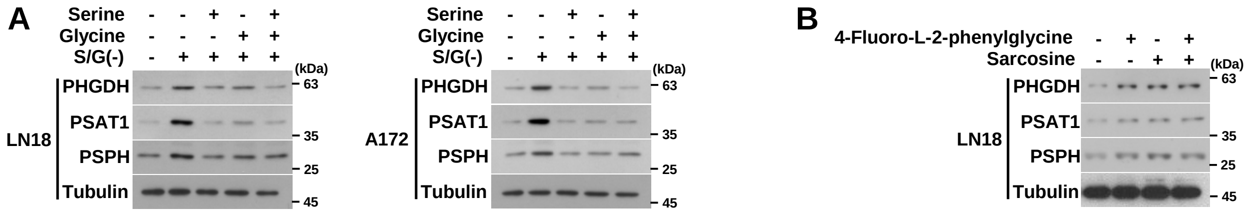
A
Serine - - + - +
Glycine - - - + +
S/G(-) - + + + +
(kDa)
LN18
PHGDH
PSAT1
PSPH
Tubulin
63
35
25
45
Serine - - + - +
Glycine - - - + +
S/G(-) - + + + +
(kDa)
A172
PHGDH
PSAT1
PSPH
Tubulin
63
35
25
45
B
4-Fluoro-L-2-phenylglycine - + - +
Sarcosine - - + +
(kDa)
LN18
PHGDH
PSAT1
PSPH
Tubulin
63
35
25
45

Figure S3

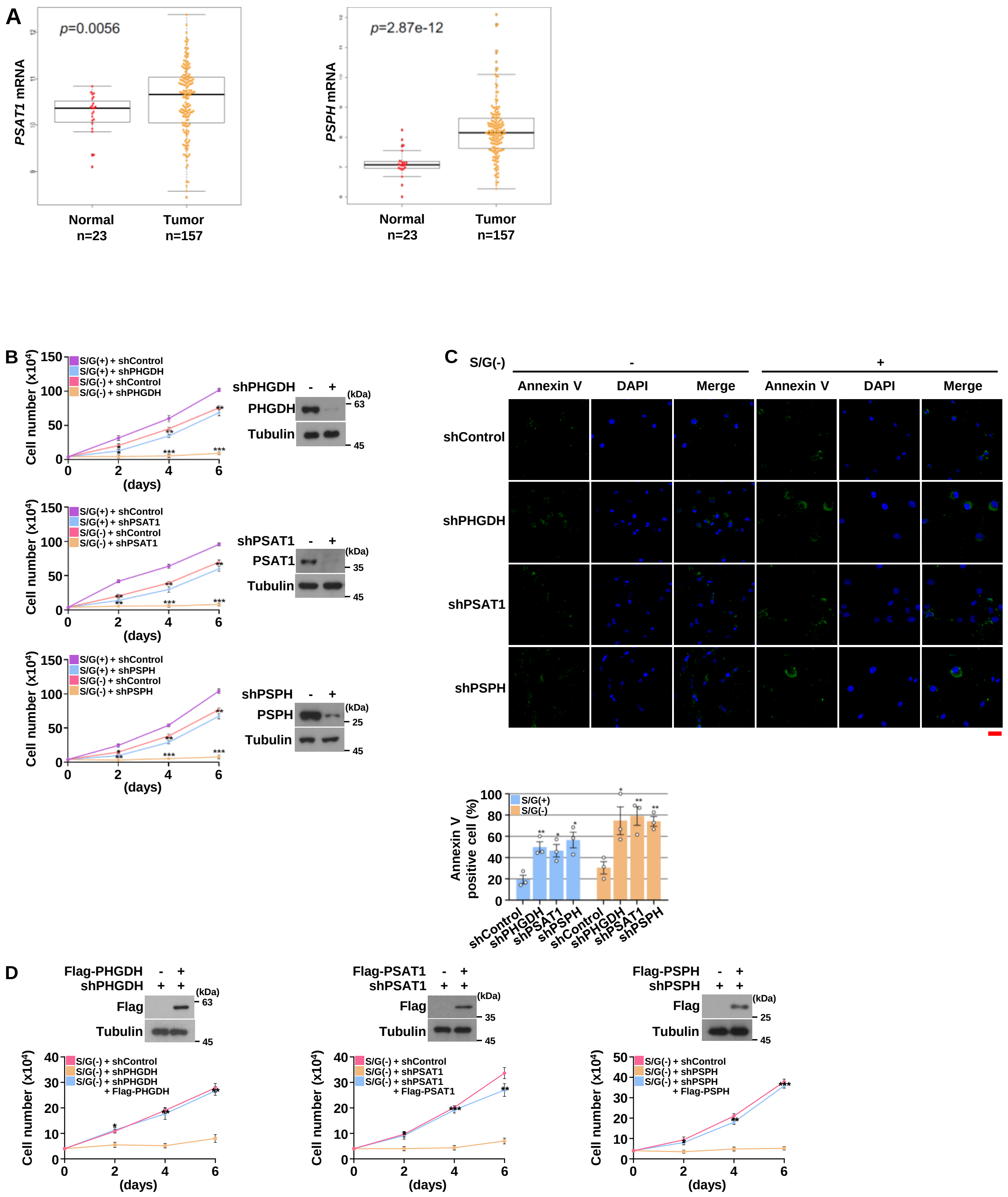

A
p=0.0056
PSAT1 mRNA
Normal n=23
Tumor n=157
p=2.87e-12
PSPH mRNA
Normal n=23
Tumor n=157
B
S/G(+) + shControl
S/G(+) + shPHGDH
S/G(-) + shControl
S/G(-) + shPHGDH
Cell number (x10⁴)
(days)
shPHGDH - +
PHGDH
Tubulin
(kDa)
63
45
S/G(+) + shPSAT1
S/G(-) + shPSAT1
shPSAT1 - +
PSAT1
35
S/G(+) + shPSPH
S/G(-) + shPSPH
shPSPH - +
PSPH
25
C
S/G(-)
Annexin V
DAPI
Merge
shControl
shPHGDH
shPSAT1
shPSPH
Annexin V positive cell (%)
S/G(+)
S/G(-)
D
Flag-PHGDH - +
shPHGDH + +
Flag
Tubulin
S/G(-) + shControl
S/G(-) + shPHGDH
S/G(-) + shPHGDH + Flag-PHGDH
Flag-PSAT1 - +
shPSAT1 + +
S/G(-) + shPSAT1
S/G(-) + shPSAT1 + Flag-PSAT1
Flag-PSPH - +
shPSPH + +
S/G(-) + shPSPH
S/G(-) + shPSPH + Flag-PSPH

Figure S4

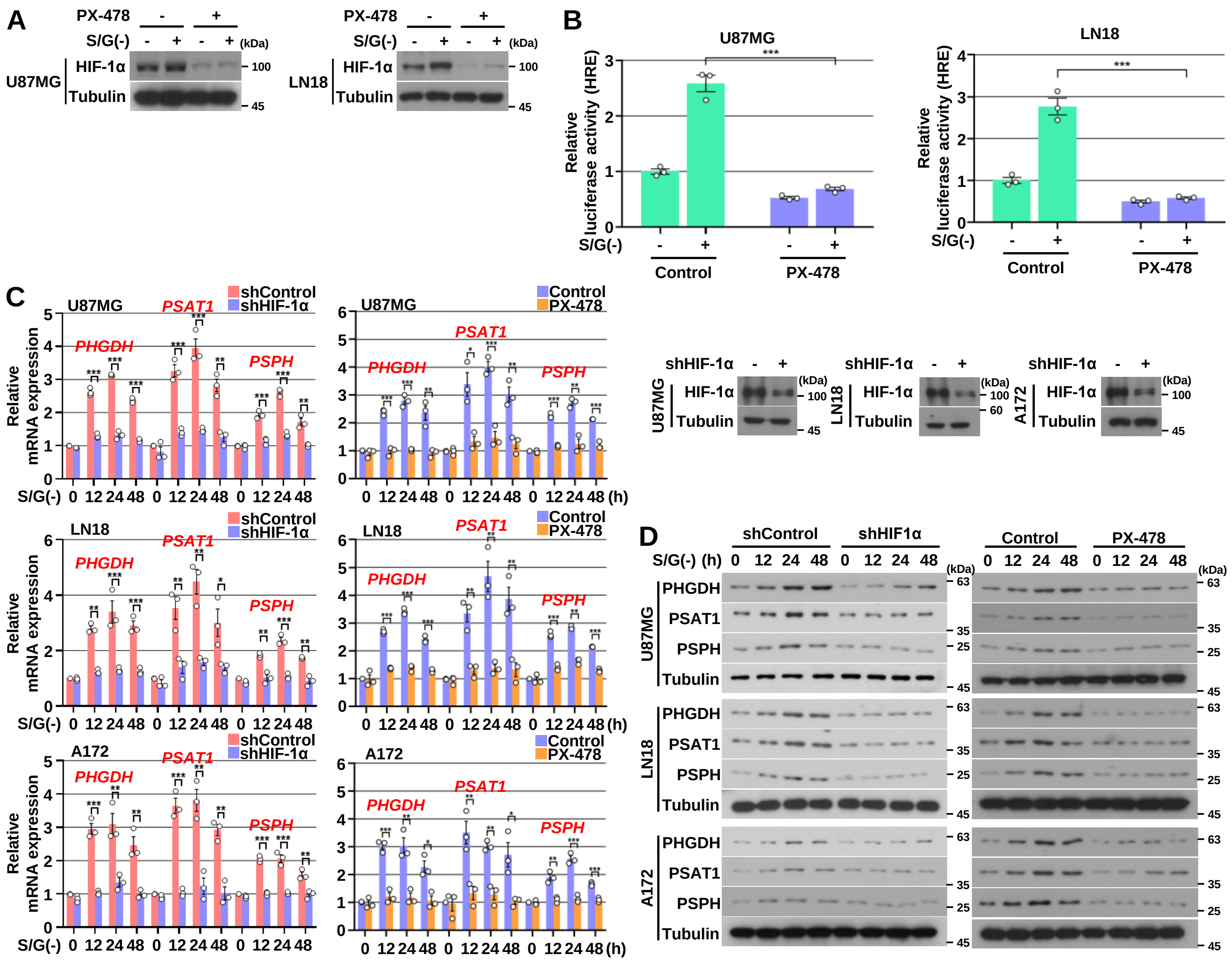
A
PX-478
S/G(-)
U87MG
HIF-1α
Tubulin
LN18
(kDa)
100
45
B
U87MG
LN18
Relative luciferase activity (HRE)
Control
PX-478
C
shControl
shHIF-1α
Control
PX-478
A172
PHGDH
PSAT1
PSPH
Relative mRNA expression
S/G(-) 0 12 24 48 0 12 24 48 0 12 24 48
0 12 24 48 0 12 24 48 0 12 24 48 (h)
shHIF-1α
60
D
shControl
shHIF1α
S/G(-) (h) 0 12 24 48 0 12 24 48
Control
PX-478
PHGDH
PSAT1
PSPH
Tubulin
63
35
25
45

Figure S5

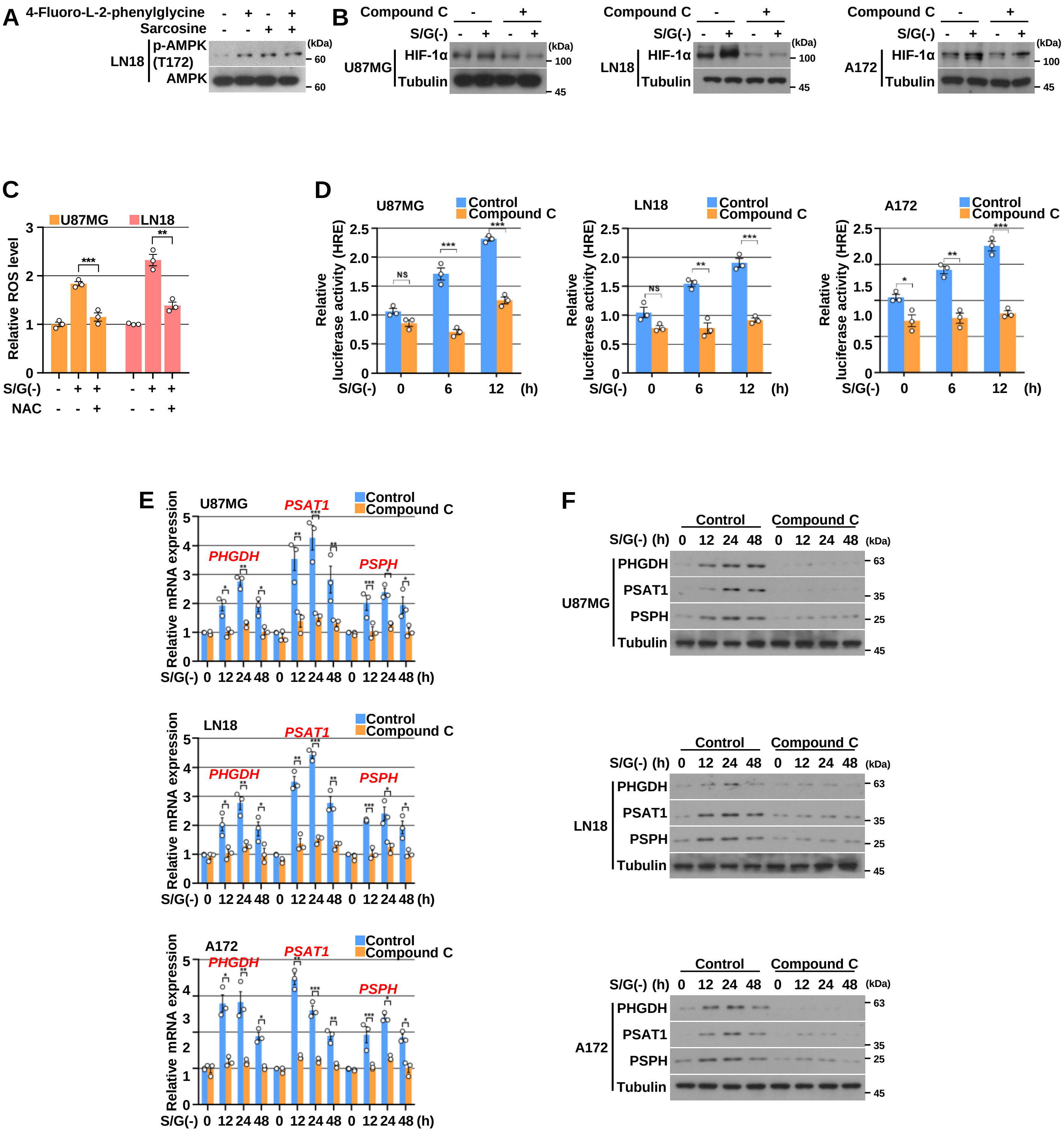

A
4-Fluoro-L-2-phenylglycine
Sarcosine
LN18
p-AMPK (T172)
AMPK
(kDa)
60
60
B
Compound C
S/G(-)
U87MG
LN18
A172
HIF-1α
Tubulin
(kDa)
100
45
C
U87MG
LN18
Relative ROS level
S/G(-)
NAC
D
Control
Compound C
Relative luciferase activity (HRE)
NS
S/G(-) 0 6 12 (h)
E
U87MG
LN18
A172
PHGDH
PSAT1
PSPH
Control
Compound C
Relative mRNA expression
S/G(-) 0 12 24 48 0 12 24 48 0 12 24 48 (h)
F
Control
Compound C
S/G(-) (h) 0 12 24 48 0 12 24 48
(kDa)
PHGDH
PSAT1
PSPH
Tubulin
63
35
25
45

Figure S6

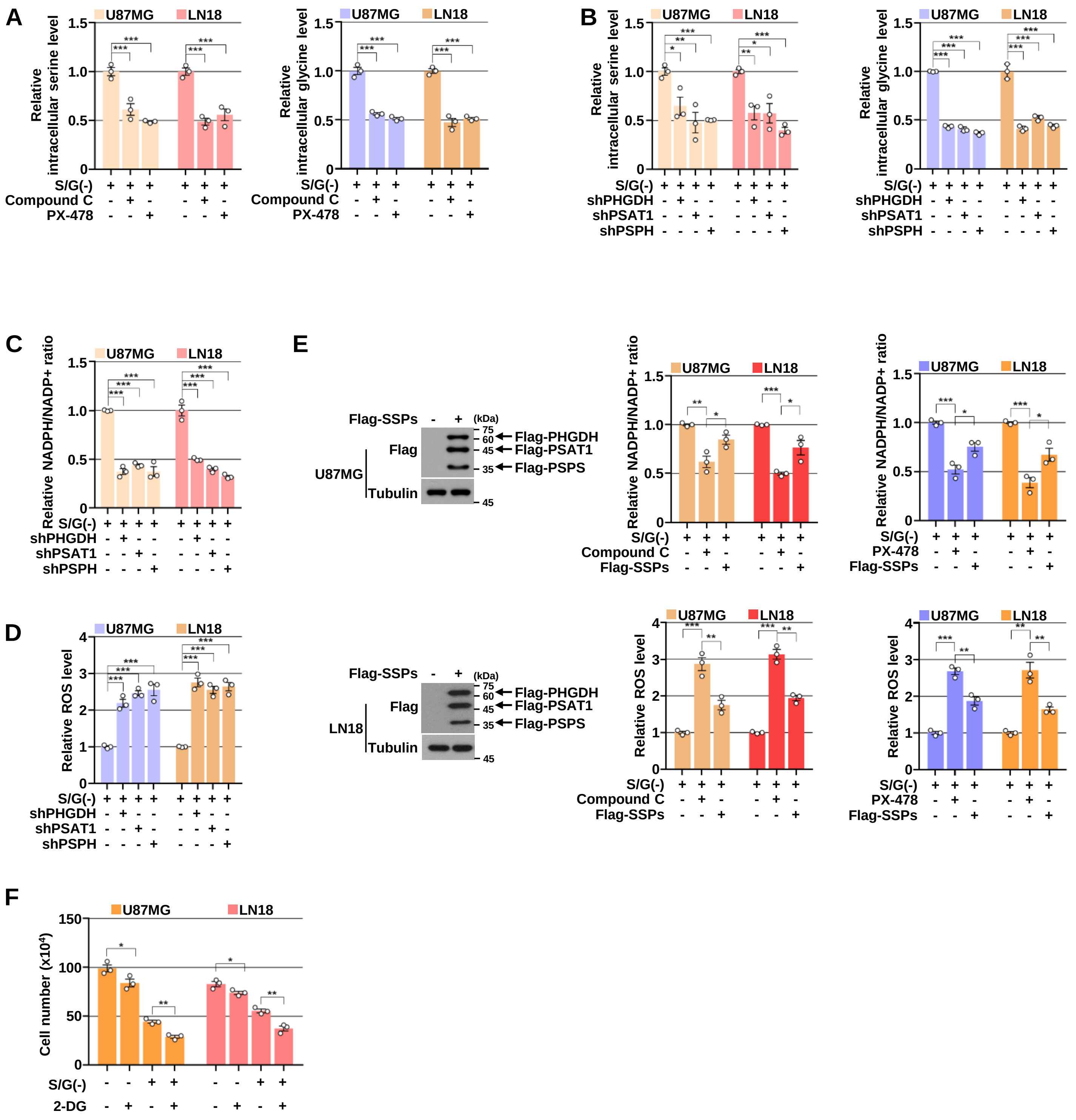
A
U87MG
LN18
Relative intracellular serine level
Relative intracellular glycine level
S/G(-)
Compound C
PX-478
B
shPHGDH
shPSAT1
shPSPH
C
Relative NADPH/NADP+ ratio
D
Relative ROS level
E
Flag-SSPs
(kDa)
Flag
Tubulin
Flag-PHGDH
Flag-PSAT1
Flag-PSPS
75
60
45
35
F
Cell number (x10⁴)
2-DG

Figure S7

Figure 2.

F

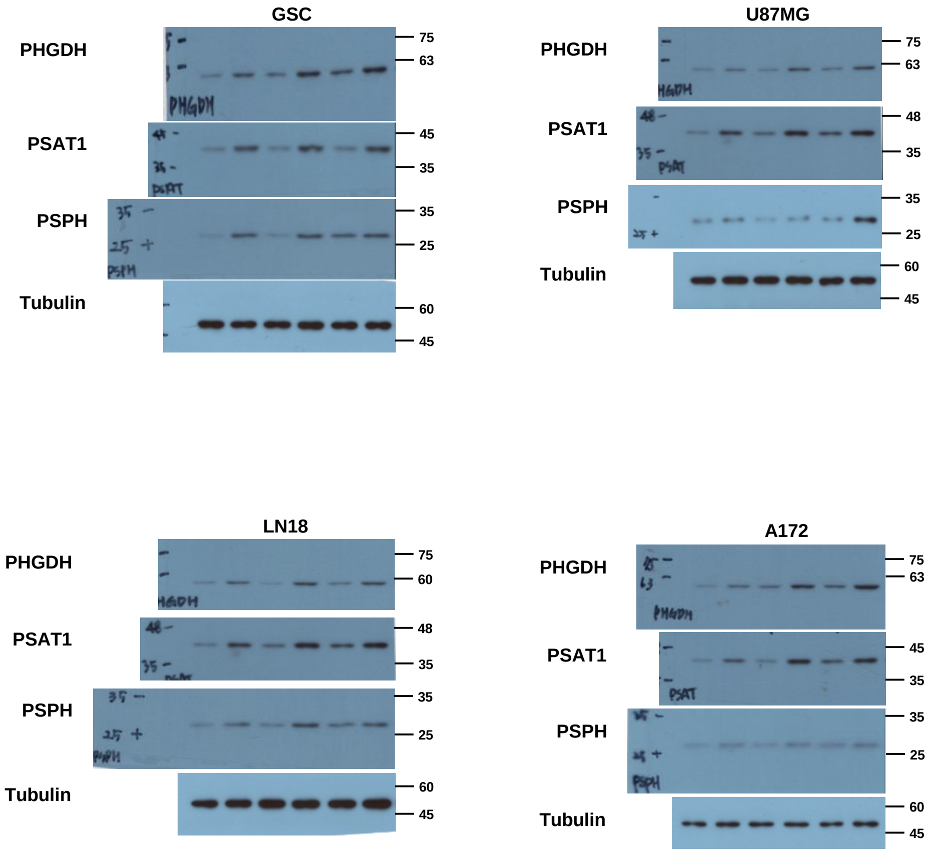

Figure 3.

B

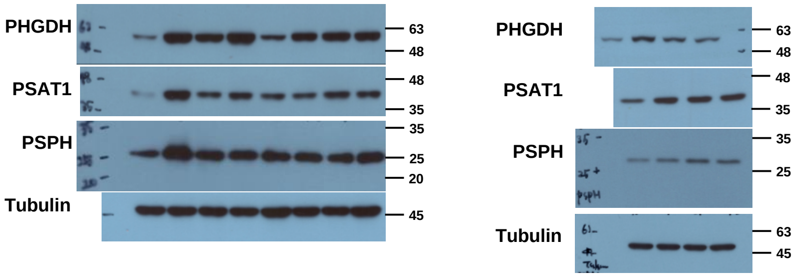

C

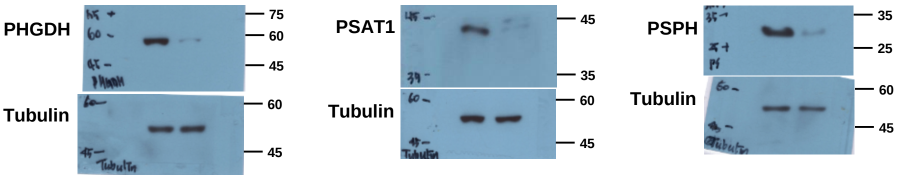

Figure 4.

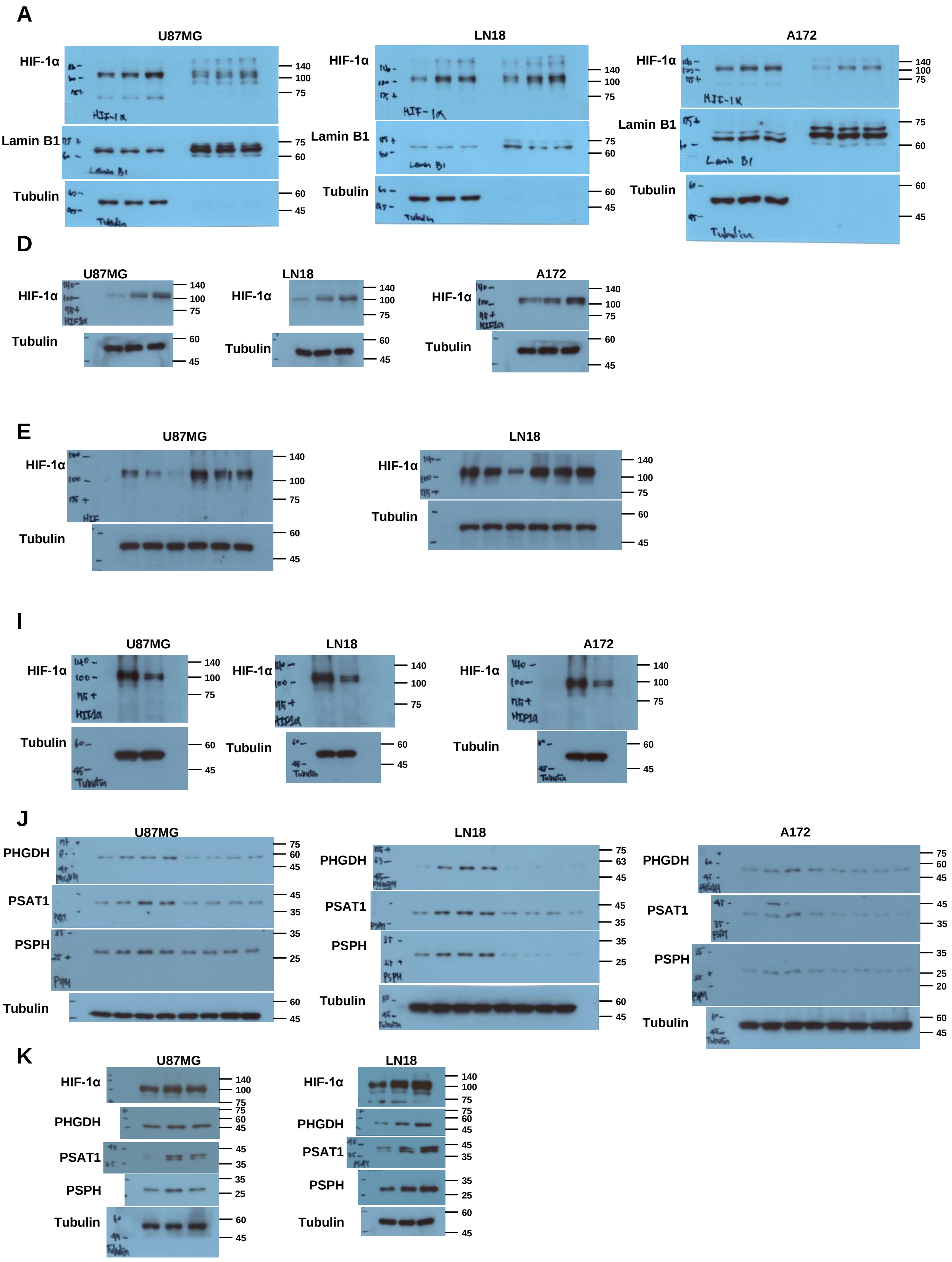
A
U87MG
LN18
A172
HIF-1α
Lamin B1
Tubulin
140
100
75
60
45
D
E
I
J
PHGDH
PSAT1
PSPH
63
35
25
20
K

Figure 5.

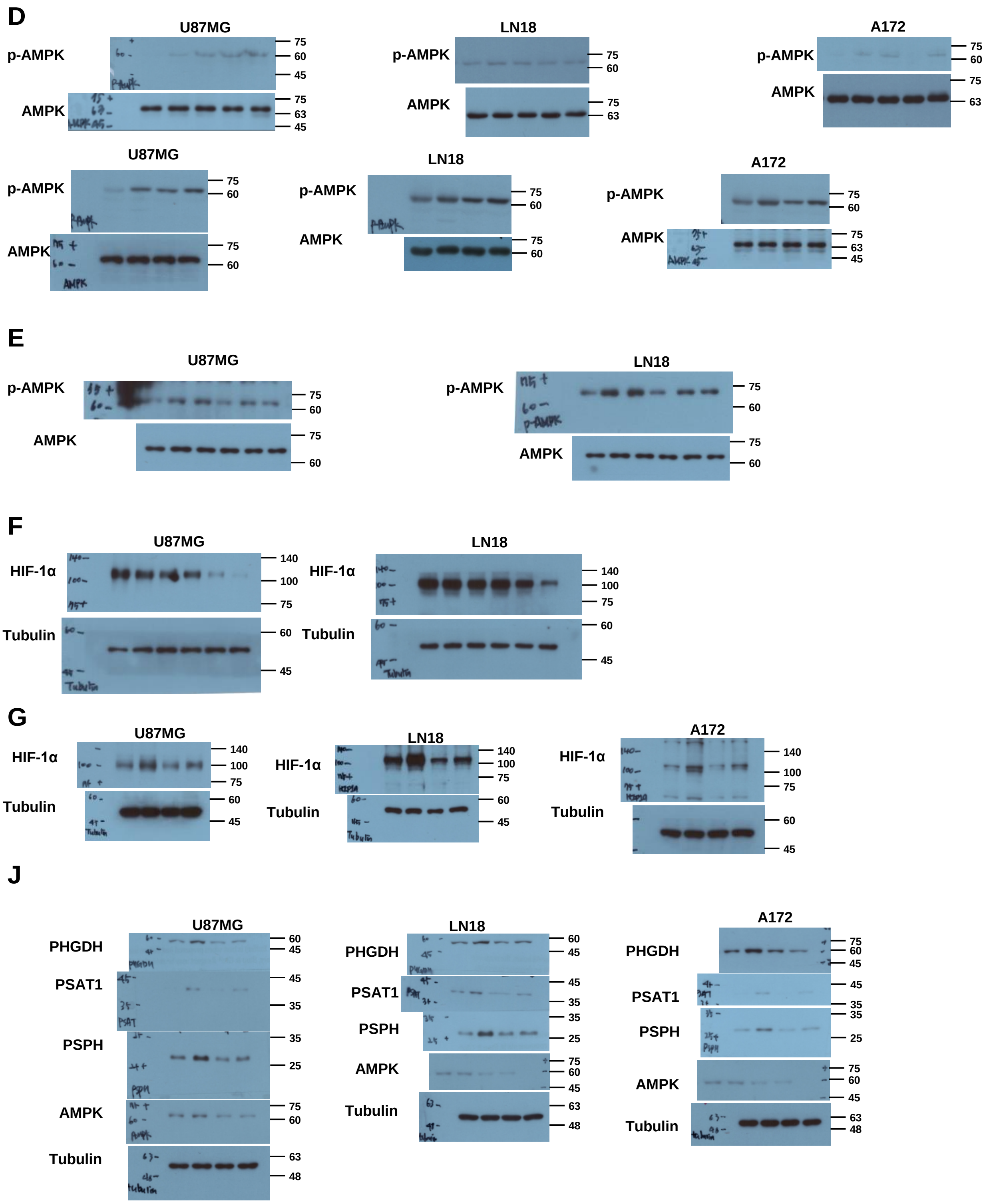
D
U87MG
p-AMPK
AMPK
75
60
45
75
63
45
LN18
p-AMPK
AMPK
75
60
75
63
A172
p-AMPK
AMPK
75
60
75
63
U87MG
p-AMPK
AMPK
75
60
75
60
LN18
p-AMPK
AMPK
75
60
75
60
A172
p-AMPK
AMPK
75
60
75
63
45
E
U87MG
p-AMPK
AMPK
75
60
75
60
LN18
p-AMPK
AMPK
75
60
75
60
F
U87MG
HIF-1α
Tubulin
140
100
75
60
45
LN18
HIF-1α
Tubulin
140
100
75
60
45
G
U87MG
HIF-1α
Tubulin
140
100
75
60
45
LN18
HIF-1α
Tubulin
140
100
75
60
45
A172
HIF-1α
Tubulin
140
100
75
60
45
J
U87MG
PHGDH
PSAT1
PSPH
AMPK
Tubulin
60
45
45
35
35
25
75
60
63
48
LN18
PHGDH
PSAT1
PSPH
AMPK
Tubulin
60
45
45
35
35
25
75
60
45
63
48
A172
PHGDH
PSAT1
PSPH
AMPK
Tubulin
75
60
45
45
35
35
25
75
60
45
63
48

Figure S2

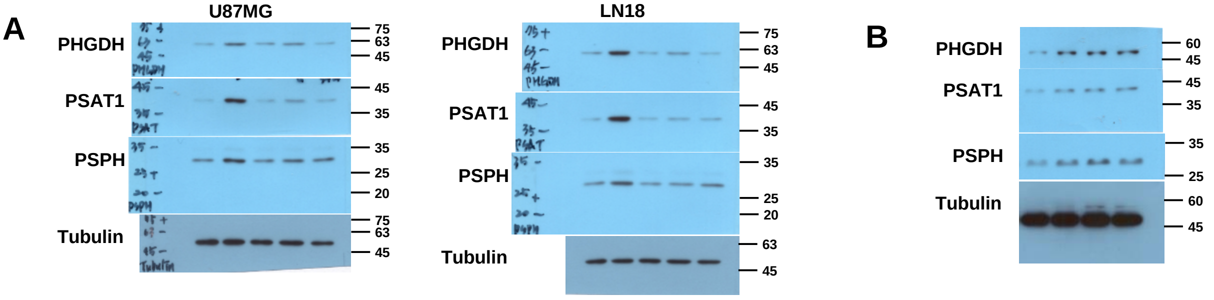

Figure S3

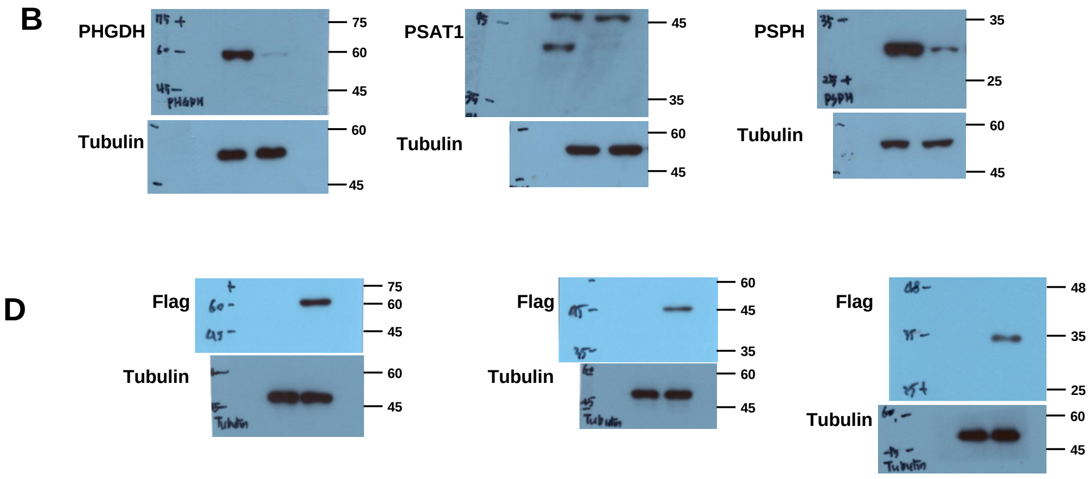

Figure S4.

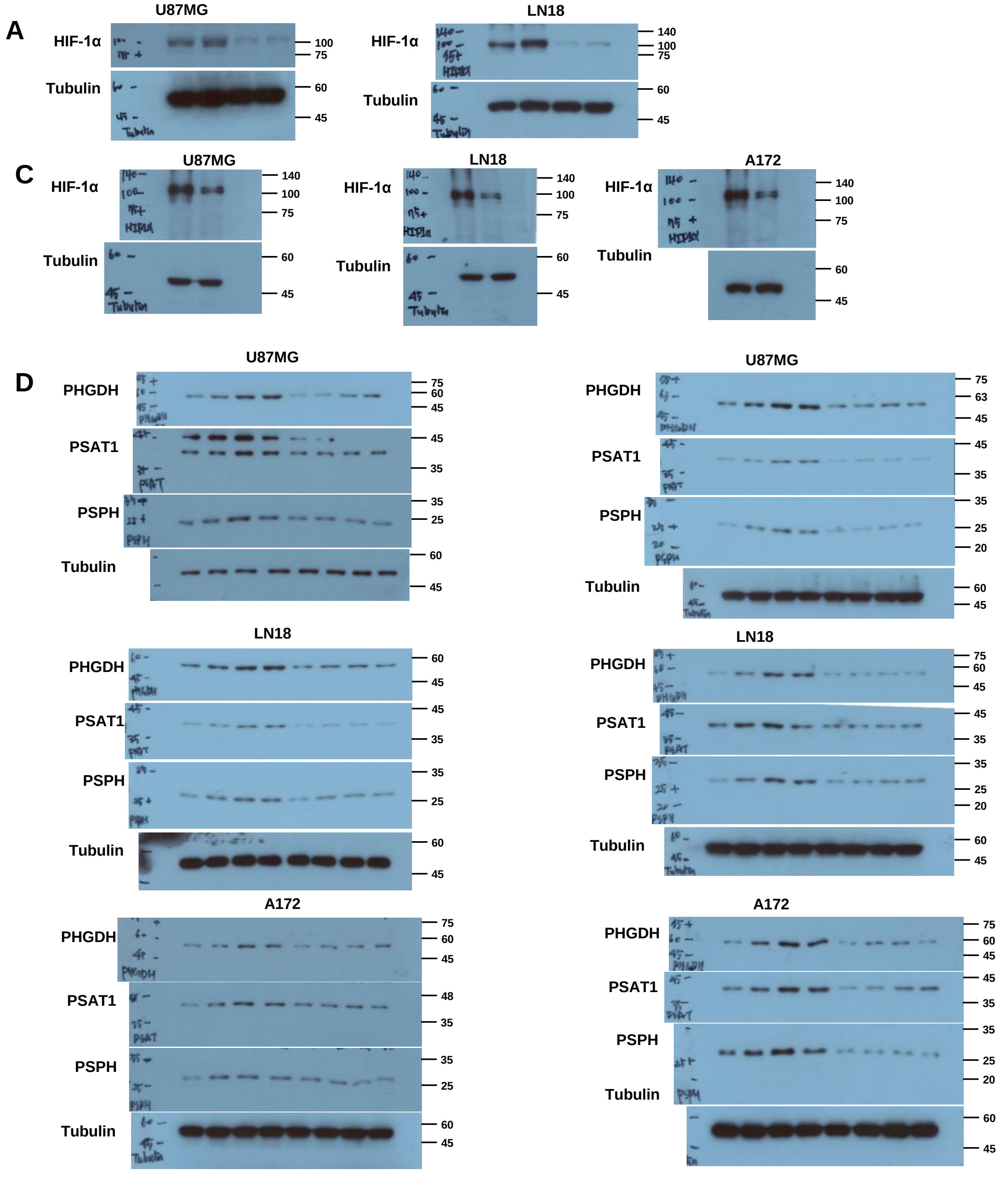
A
U87MG
HIF-1α
Tubulin
100
75
60
45
LN18
HIF-1α
Tubulin
140
100
75
60
45
C
U87MG
LN18
A172
HIF-1α
Tubulin
140
100
75
60
45
D
U87MG
LN18
A172
PHGDH
PSAT1
PSPH
Tubulin
75
63
60
48
45
35
25
20

Figure S5

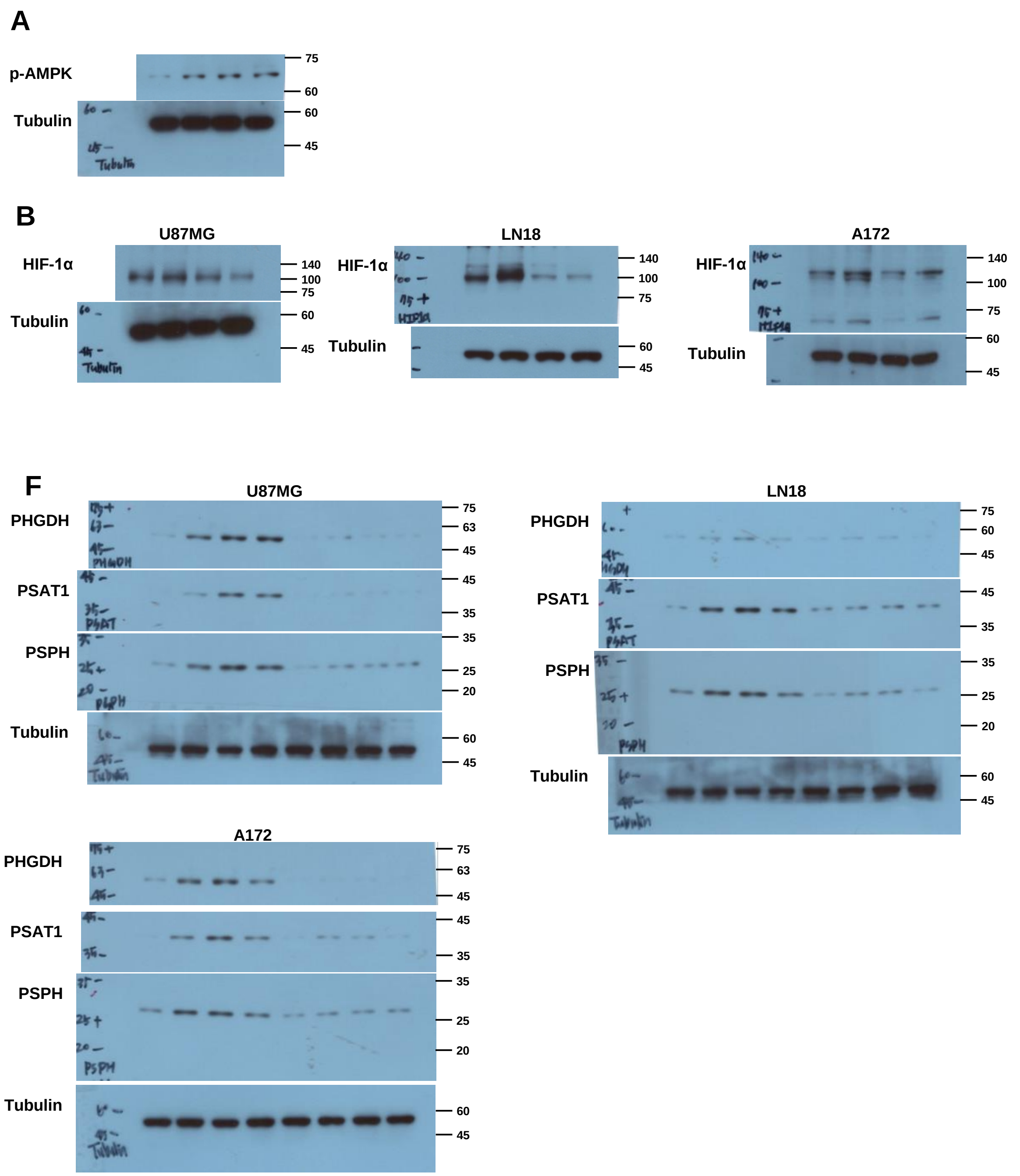

Figure S6

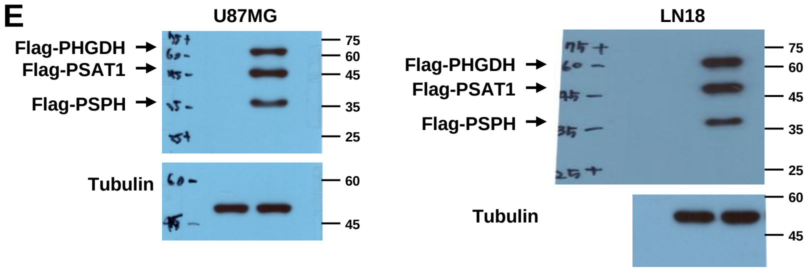

Supplement: Supplementary file 1 — Additional file 1: Table S1. Enriched top 10 gene ontology annotations for biological process in transcriptome of S/G-deprived U87MG cells, compared to control. Table S2. Enriched top 10 gene ontology annotations for molecular function in transcriptome of S/G-deprived U87MG cells, compared to control. Table S3. Enriched top 10 gene ontology annotations for cellular component in transcriptome of S/G-deprived U87MG cells, compared to control. Table S4. Differentially expressed transcriptome of S/G-deprived U87MG cells, compared to control, in trimmed IPA network. Figure S1. (related to Figure 1). DEGs network analyses of transcriptome from U87MG cells in response to serine/glycine deprivation. Figure S2. (related to Figure 2). Serine/glycine deprivation induces glucose uptake, glycolytic flux, and de novo serine biosynthesis. Figure S3. (related to Figure 3). Serine synthesis pathway genes are overexpressed in gliomas and required for brain tumor growth. Figure S4. (related to Figure 4). HIF-1α induces SSP gene expression in response to 3 serine/glycine deprivation. Figure S5. (related to Figure 5). AMPK activation is required for the HIF-1α-induced SSP gene expression in response to serine/glycine deprivation. Figure S6. (related to Figure 6). AMPK-HIF-1α signaling promotes de novo serine biosynthesis, proliferation, and survival of GBM cells upon serine/glycine deprivation. Figure S7. Full uncut blots are presented for the representative western blots featured in the present study. [file 13046_2023_2927_MOESM1_ESM.pdf]
